# Quantitative MRI of the hippocampus reveals microstructural trajectories of aging and Alzheimer’s disease pathology

**DOI:** 10.1073/pnas.2502674122

**Published:** 2025-10-27

**Authors:** Alfie Wearn, Christine L. Tardif, Ilana R. Leppert, Giulia Baracchini, Colleen Hughes, Jennifer Tremblay-Mercier, John Breitner, Judes Poirier, Sylvia Villeneuve, Boris C. Bernhardt, Gary R. Turner, R. Nathan Spreng, the PREVENT-AD Research Group

**Affiliations:** ^a^Montreal Neurological Institute, Department of Neurology and Neurosurgery, McGill University, Montreal, QC H3A 2B4, Canada; ^b^McConnell Brain Imaging Centre, McGill University, Montreal, QC H3A 2B4, Canada; ^c^Douglas Mental Health University Institute, Verdun, QC H4H 1R3, Canada; ^d^Departments of Psychiatry, McGill University, Montreal, QC H3A 1A1, Canada; ^e^Department of Psychology, York University, Toronto, ON M3J 1P3, Canada; ^f^Department of Psychology, McGill University, Montreal, QC H3A 3E8, Canada

**Keywords:** hippocampus, aging, Alzheimer’s disease, MRI, microstructure

## Abstract

Hippocampal degeneration is a feature of both normal aging and Alzheimer’s disease (AD). Prior to macroscopic degeneration, microstructural changes occur such as demyelination, iron deposition, or subtle atrophy, which can be characterized in vivo using MRI. We topographically mapped measures of microstructure and macrostructure across the unfolded surface of the hippocampus in 224 healthy older adults at risk for AD (aged 57 to 87) and 37 younger adults (aged 18 to 37). We describe three spatial regions of unique structural covariance between four parameters sensitive to microstructural tissue properties (R1, MTsat, R2*, PD) and macrostructure (surface thickness), with high convergence with previous spatial segmentations. We demonstrate both cross-sectional and longitudinal associations of microstructure with healthy aging across the lifespan, AD pathological hallmarks, genetic risk, and cognition. These associations had subtle variations across different spatial areas of the hippocampus. We report associations between age and qMRI measures sensitive to macromolecular concentration (R1, MTsat) and paramagnetic susceptibility (R2*), consistent with mechanisms of demyelination and increased iron deposition as key hallmarks of the aging hippocampus and in presymptomatic stages of AD. qMRI measures did not explain more variance in delayed recall than global PET measures, suggesting variation in cognitive performance in aging and incipient AD is influenced by factors beyond hippocampal microstructure. We demonstrate the utility of quantitative MRI to provide greater insight into hippocampal health compared to typical macrostructural measures through “in vivo histology,” opening a window to understanding neuropathological mechanisms in the earliest stages of age- and disease-related neurodegeneration.

## Significance

Hippocampal atrophy, typically measured using volumetry, is a hallmark feature of both normal aging and Alzheimer’s disease (AD). However, the earliest stages of atrophy manifest as microstructural changes in tissue composition rather than macroscopic volume loss. We conducted longitudinal in vivo mapping of hippocampal microstructure in healthy aging and incipient AD, highlighting demyelination, iron deposition, and changes in water content as markers of age and AD risk. A combination of macrostructural and microstructural measures provides a more comprehensive picture of brain health and disease, unlocking unique insights into the pathological state of brain tissue and the impact of AD at a point where therapeutic rescue of the tissue is most likely to be efficacious.

The burden of age-related disorders on society will worsen significantly in coming years due to the increasing proportion of older adults in the population (1). Age is a risk factor for many chronic disorders, of which Alzheimer’s disease (AD) is both one of the most common and least treatable (2). AD is a disorder distinct from normal aging but both trajectories feature progressive neurodegeneration resulting in cognitive decline. Particularly notable changes are well reported within the hippocampus, in both aging and AD (3–5). A better understanding of how the hippocampus is affected by age and AD pathology is critical if we are to effectively disentangle trajectories of AD from healthy aging, and to identify targets for therapies that slow or prevent irreversible neurodegeneration and resultant cognitive decline. Quantitative MRI (qMRI) provides the means to characterize the health of the living human hippocampus through a method sometimes referred to as “in vivo histology” (6, 7).

Hippocampal atrophy, often quantified using volumetry, is observed in older age (8, 9) and is a key hallmark of AD (10–12). Earlier hippocampal changes due to aging and neurodegenerative disease are characterized by subtle changes in tissue composition and biophysical environment such as demyelination (13, 14), elevated iron (15, 16), edema (17), or inflammation (18–20). These microstructural changes have classically been studied through tissue histology, with different stains highlighting different tissue components. qMRI allows us to collect noninvasive biologically interpretable measures of different elements of tissue microstructure from living tissue (21–24). Similar to the choice of tissue stains in histology, one can derive measures from a number of MRI parameters with unique sensitivities to the biophysical environment of a given tissue to provide more information than typical macrostructural imaging. For this reason, the term “in vivo histology” has been used to describe this technique (22). For example, magnetization transfer saturation (MTsat) provides a quantification of tissue macromolecules, and correlates strongly with myelin content (25, 26). Effective transverse relaxation rate (R2*) has shown strong correlations with iron as well as sensitivity to myelin (24, 27, 28). Proton density (PD) is a sensitive measure of the water content within a tissue (29, 30). The longitudinal relaxation rate (R1) is sensitive to a combination of tissue physicochemical environment, myelin, water, and iron (31). Assessing multiple measures simultaneously, accommodated through multiparametric mapping sequences (32–34), also provides a more complete picture of the underlying causes of signal changes as multiple simultaneously collected parameters can be competitively assessed to increase biophysical specificity. Furthermore, given the necessarily cross-sectional nature of postmortem histological studies, these measures provide a unique opportunity to longitudinally track microstructural changes in the brain within individuals. In short, in vivo qMRI provides an unparalleled means to study brain health and disease and promises to provide valuable insights at a time when clinical intervention to arrest neurodegeneration is most likely to be clinically effective. Recent efforts have mapped qMRI measures to the hippocampus in young adults (6, 7), but lifespan patterns, associations to AD pathology, and longitudinal trajectories remain to be determined. Here, we use qMRI to describe in vivo mapping of hippocampal microstructure in healthy aging and AD in humans in a longitudinal cohort study.

Although often thought of as a subcortical structure due to its location deep with the medial temporal lobe, the adult hippocampus comprises two folded allocortical ribbons, one of which (the cornu Ammonis, CA) is topographically contiguous with the parahippocampal and entorhinal neocortices (35, 36). The cytoarchitectonic organization of the hippocampus varies along the length of this mantle. The subiculum lies topographically adjacent to the entorhinal cortex at the most distal end, which, moving proximally along the mantle, becomes the CA regions 1 to 4, consecutively. Most proximally, in a topographically distinct mantle that separates from the CA during development, lies the dentate gyrus (DG) (35). As well as distinct cytoarchitectonic morphology, these subfields have varying vulnerabilities to age-related pathological damage, with subiculum and CA1 (distal regions) expressing greater vulnerability and DG (proximal regions) remaining resistant for longer (37, 38). These cytoarchitectonic subfields are defined primarily along this proximal–distal axis; however, there is also considerable structural and functional specialization as well as variation in gene expression along the anterior–posterior “long-axis” (39–43). The most popular definition of distinct subregions along this axis is the “tripartite” model, which splits the hippocampus into head (most anterior), body, and tail (most posterior) (42, 44).

In this study, we leverage advances in qMRI multiparametric mapping, which allows for rapid simultaneous acquisition of multiple parameters sensitive to microstructural features (21, 33). We examine longitudinal trajectories of hippocampal microstructure using state-of-the-art machine learning techniques designed to map parameters to the hippocampal allocortical surface (45). Across the hippocampus and its different data-defined spatial subregions, we: 1) compared hippocampal macro- and microstructure between older and younger cohorts of cognitively healthy adults, 2) examined cross-sectional relationships with age within both age groups and 3) assessed longitudinal trajectories of structure. Our cohort of older adults all had a first-degree family history of AD. The group was thus enriched for individuals in the preclinical stages of AD, which provided a unique opportunity to 4) examine cross-sectional and longitudinal relationships between hippocampal structure and markers of AD pathology. Additionally, we 5) examined cross-sectional and longitudinal differences between subjects with and without an APOE4 allele, a well-characterized AD risk gene (46). Finally, we 6) assessed the extent to which hippocampal structure explained individual differences in cognitive performance. Across these analyses we expected measures of microstructure to provide greater insight into hippocampal health than measures of macrostructure, in particular, providing greater sensitivity to variation in preclinical AD pathology.

## Results

### Study Participants.

Hippocampal structure was mapped in a total of 261 individuals. This includes 224 older adults with familial risk for AD (mean age at baseline 68.7 ± 5.43 y, range: 57.2-87.7, female: 73.2%) and 37 younger adults (mean age 24.4 ± 4.74 y, range: 18.5-37.9, female: 56.8%). 143 older adults had an additional timepoint, including one participant with three total timepoints. The mean (±SD) time between sessions was 32.0 ± 5.22 mo. All individuals underwent identical 3 T MRI scanning procedures. Only older adults received PET scans and only older adult data for cognitive testing were examined.

### Mapping Microstructure and Macrostructure of the Hippocampus.

#### Vertex-wise microstructure mapping.

**Fig. 1.**
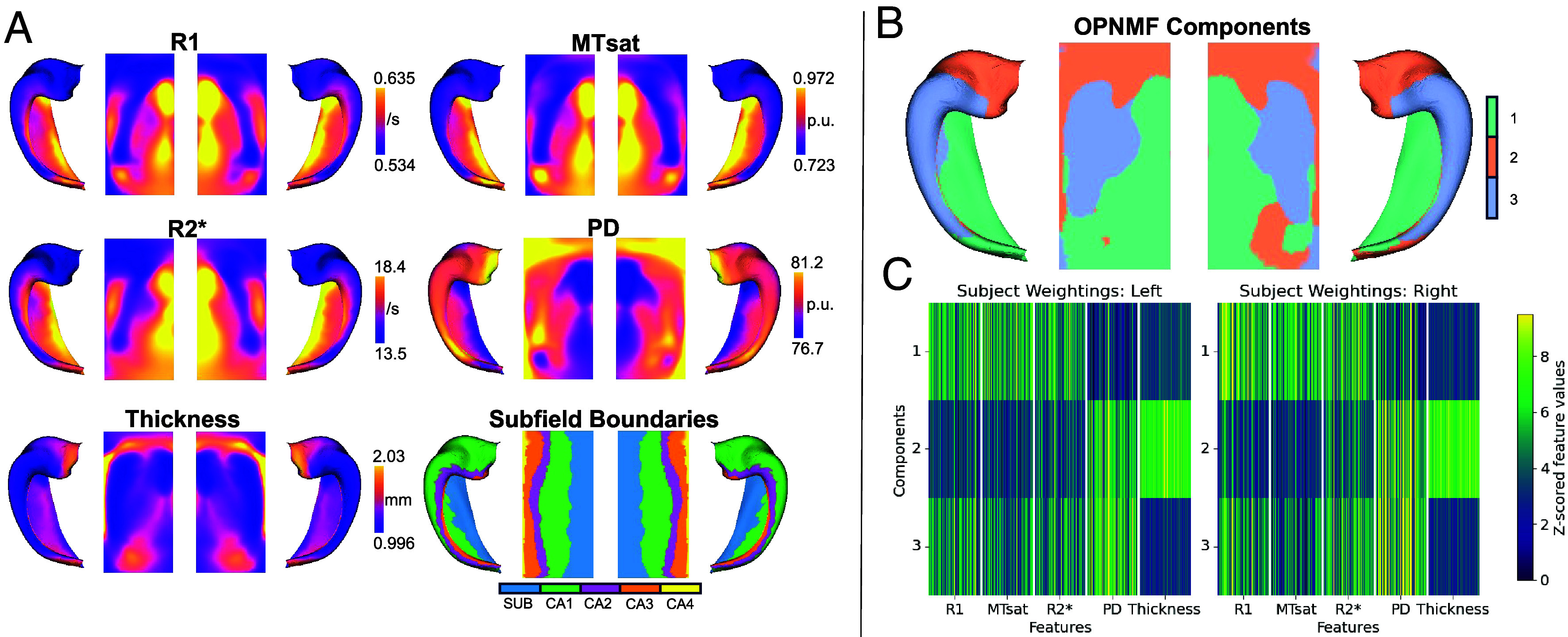
Mapping structural measures to the hippocampal surface. (*A*) Heat maps for each of the five measures of structure mapped to the folded and unfolded surfaces of bilateral hippocampus, averaged across all subjects. Cytoarchitectonic subfield boundaries are also shown for reference. p.u.= percentage units. (*B*) OPNMF-derived spatial component boundaries. (*C*) OPNMF subject weightings. Each row corresponds to a spatial component in *B*. Each column represents a single structural value for a single subject. White bars separate structural measures. Values are z-scored within each structural measure and component.

The hippocampus comprises a folded ribbon of allocortex that can be computationally unfolded into a 2D surface using a novel tool, HippUnfold (45). Four quantitative MRI-derived microstructural measures (R1, MTsat, R2*, and PD) as well as a macrostructural measure (hippocampal surface thickness) were mapped onto each vertex of the hippocampal surface, shown as a cohort average in Fig. 1*A*.

#### Orthogonally projected nonnegative matrix factorization.

Neuroimaging studies of hippocampal subfields have provided considerable information about age- and disease-related pathophysiology (11, 12, 47–52). However, typical resolutions and contrasts of human neuroimaging do not allow for direct imaging of the borders between these cytoarchitectonic boundaries, leading to variability between segmentation protocols (53). Even as consensus is reached on boundary definition on MRI thanks to the work of the Hippocampal Subfields Group (54), many border placements rely on geometric rules which may not apply in atypical aged or diseased hippocampi. Data-driven methods such as orthogonally projected nonnegative matrix factorization (OPNMF) have been used in several recent studies to identify spatially contiguous regions of macro- and microstructural covariance in the brain (6, 7, 55, 56).

Through stability and accuracy assessment, we determined that a three-component solution was optimal with our chosen parameters (*SI Appendix*, Fig. S1). The three identified components respectively represent areas of 1) high R1/MTsat/R2* and low PD/thickness, 2) low R1/MTsat/R2* and high PD/thickness and 3) high PD and low thickness (with middling R1/MTsat/R2*). The relative contribution of each macro- or microstructural feature to each component is shown in Fig. 1*C*. These three components, shown in Fig. 1*B*, respectively cover 1) the posterior-distal surface, corresponding to the hippocampal tail and subiculum and partial-CA1 in the body and tail, 2) the hippocampal head as well as a smaller subregion of hippocampal tail, and 3) the proximal surface along body and tail, corresponding to the CA1-3 along the superior edge of the folded hippocampus. Average structural values for each spatial component, and values of regional dispersion, are shown in *SI Appendix*, Table S1.

### Trajectories of Macro- and Microstructure Across the Lifespan.

**Fig. 2.**
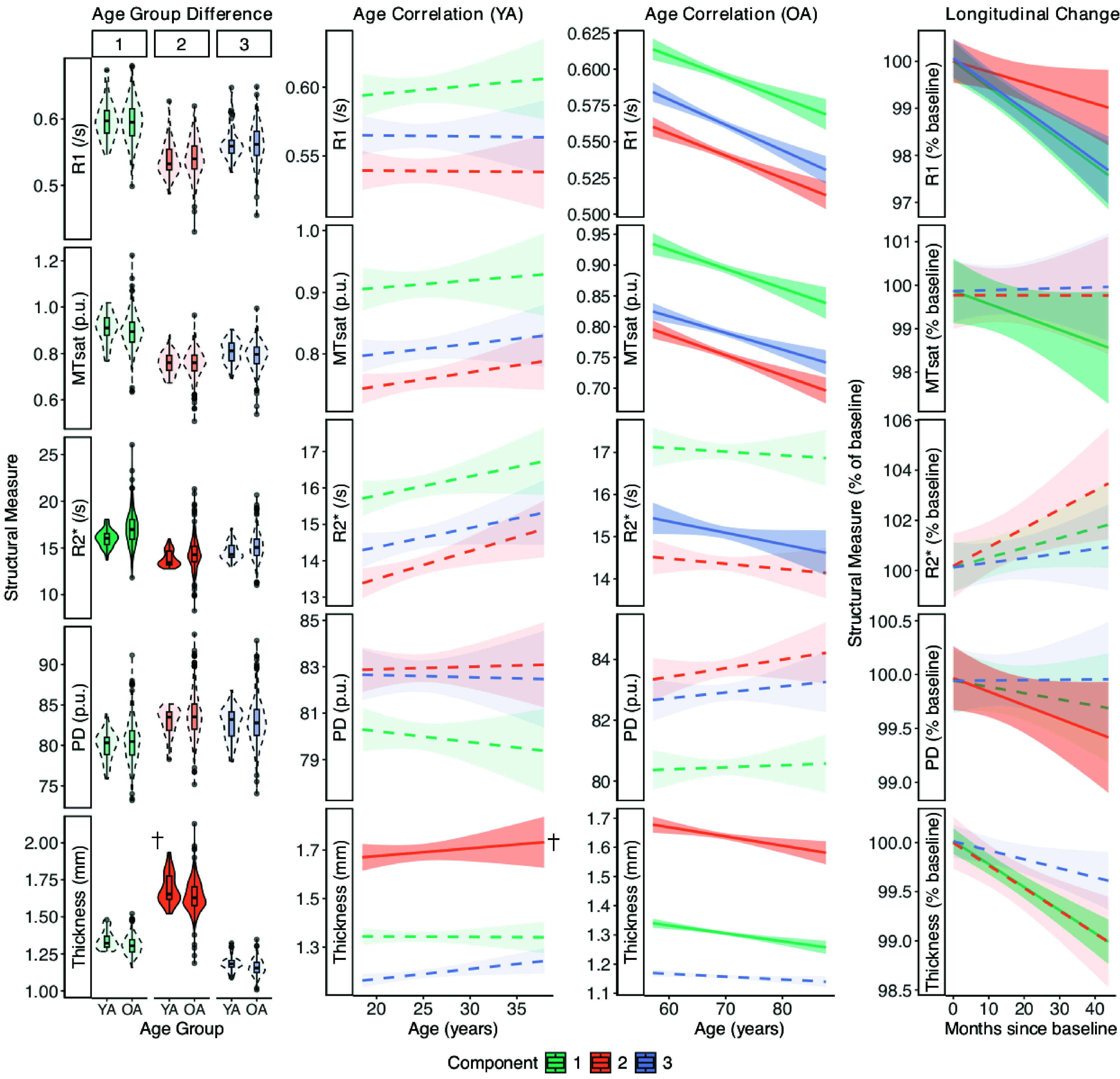
Characterizing cross-sectional and longitudinal lifespan effects of hippocampal macro- and microstructure. Each row corresponds to a measure of structure, with each column representing a main effect from the mixed effect models in which effects were allowed to vary by each component. Shaded and solid plots represent significant component effects while faded and dotted plots represent nonsignificant effects, with a dagger (†) indicating effects that do not survive correction for multiple comparisons. Data within each plot are partial-residuals, meaning they are corrected for other variables in the model. For clarity, regression plots are shown as mean ± SE summary lines. Full data distributions are shown in *SI Appendix*, Fig. S2. YA = Younger adults, OA = Older adults.

Our primary questions of interest were whether there was any relationship between age and our measures of hippocampal structure, and whether any change over time within a given measure was observed. To assess these effects, a linear mixed-effects model was run for each of the five structural measures. We also tested spatial specificity of all effects, with a separate model in which OPNMF-derived spatial components were examined (Fig. 2). The main effects tested were as follows: 1) cross-sectional age group differences (younger vs older); 2) young adult cross-sectional age effects; 3) older adult cross-sectional age effects; 4) longitudinal change within older adults. These models are described in full in the *SI Appendix* (SI Methods: *Statistical Analysis*) and statistics shown in Table 1 (whole hippocampus statistics) and *SI Appendix*, Table S2 (including component-specific statistics).

**Table 1.** Statistics for models characterizing cross-sectional and longitudinal lifespan effects of hippocampal macro- and microstructure

	Age group			Age (Young)			Age (Older)			Longitudinal change		
	*t*-value	*P*-value	df	*t*-value	*P*-value	df	*t*-value	*P*-value	df	*t*-value	*P*-value	df
R1	0.67	0.501	278	1.21	0.228	278	**−6.64**	**0.000**	**253**	**−7.55**	**0.000**	**2270**
MTsat	−1.42	0.157	287	0.76	0.450	287	**−5.13**	**0.000**	**254**	**−3.25**	**0.001**	**2300**
R2*	**3.45**	**0.001**	**292**	1.39	0.167	292	1.24	0.216	257	**2.21**	**0.027**	**2300**
PD	0.72	0.472	266	0.23	0.821	266	0.06	0.953	254	**−3.25**	**0.001**	**2220**
Thickness	−1.89	0.060	313	1.93	0.055	314	**−3.70**	**0.000**	**258**	**−2.97**	**0.003**	**2350**

The results for the whole hippocampal models are shown here with component-specific effects in *SI Appendix*, Table S2. *P* < 0.05 effects are shown in bold. *P*-values calculated from robust model *t*-values and standard model degrees of freedom (df).

R1, MTsat, and PD were not significantly different between younger and older adult groups at their respective mean ages. However, compared to younger adults, older adults had significantly greater hippocampal R2*. This effect was statistically significant within spatial components 1 and 2 of the hippocampus. In our whole hippocampal analysis, older adults were not significantly different from younger adults in hippocampal thickness. We did observe thinner hippocampal surfaces in older compared to younger adults specific to component 2 however this effect did not survive correction for multiple comparisons.

Within our younger adult group (age 18.5 to 37.9 y), no association with chronological age was observed across the whole hippocampus for any measure. However, we did observe a positive correlation between cross-sectional age in this group and thickness of component 2.

Within our older adult group (age 57.2 to 87.7 y), older age was strongly associated with lower R1, lower MTsat, and lower thickness across the whole hippocampus. Similar associations for R1 and MTsat were observed for all individual spatial components, indicating no single driving component, but for thickness the effect was only observed in components 1 and 2. A significant negative correlation was observed in the older adults between age and R2* that was specific to component 3.

Longitudinal change was observed for all measures of microstructure and thickness. R1 decreased over time across the whole hippocampus and in all individual components. MTsat also decreased over time across the whole hippocampus driven by component 1. R2* increased over time within individuals across the whole hippocampus, however we did not observe a significant change in R2* over time in any single component. A decline in PD over time was observed across the whole hippocampus, driven by component 2. Hippocampal thickness decreased over time across the whole hippocampus driven by component 1.

Results for the main effect of sex are shown in supporting information (*SI Appendix*, Table S6).

### AD Pathology Is Associated With Cross-Sectional Variation and Longitudinal Changes in Hippocampal Microstructure.

**Fig. 3.**
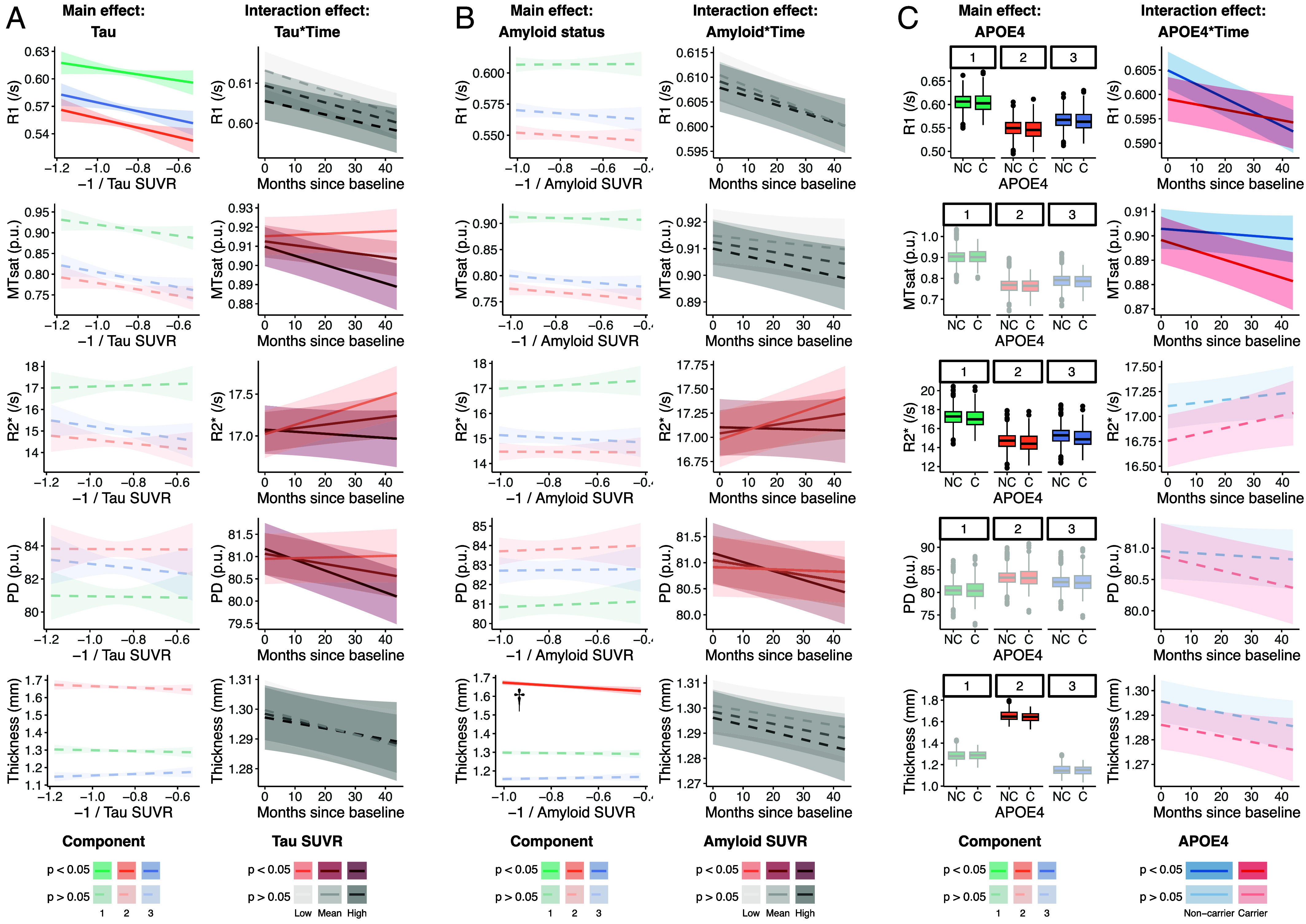
Characterizing cross-sectional and longitudinal associations between hippocampal structure and (*A*) Tau load, (*B*) Amyloid load and (*C*) APOE4 carrier status. Each row corresponds to a structural measure. Each column represents a main effect from the mixed-effects models. For each of *A*–*C*, the *Left* column shows the main cross-sectional effect, and the *Right* column shows the interaction between that main effect and time. Faded and dotted plots represent nonsignificant effects. For clarity, longitudinal effects are not shown for each component but represent the whole hippocampal model results. Data on the y-axis within each plot are partial-residuals, meaning they are corrected for other variables in the model. APOE4 plot x-axes labels: C = carrier, NC = noncarrier. In SUVR legends, Low and High indicate 1 SD below/above the mean, respectively. Effect marked with a dagger (†) was not significant after correcting for multiple comparisons. For clarity, regression plots are shown as mean ± SE summary lines. Full data distributions are shown in *SI Appendix*, Fig. S2. Semiquantitative measures (MTsat and PD) are expressed in percentage units (p.u.).

We next tested for associations between hippocampal structure and AD pathology in our older adults, using both tau and amyloid PET (Fig. 3). For tau, we calculated a voxel-number-weighted mean of Standardized uptake value ratio (SUVR) within (primarily temporal) brain regions known to be affected early by AD, previously referred to as a “meta ROI” (57). For amyloid, an “amyloid index” score was used that summarizes amyloid load within cortical regions (all regions for both meta-ROI and amyloid index are listed in the *SI Appendix* (SI Methods). We tested whether associations were present between pathology and structure at baseline, and whether longitudinal change in structure was associated with different levels of AD pathology, correcting for age, sex, and years of education. Associations were tested across the whole hippocampus and within individual spatial components. 190 older adults had PET data. 149 of these had multiple MRI timepoints. Full statistics for these models are shown in Table 2 (whole hippocampus statistics) and *SI Appendix*, Table S3 (including component-specific statistics).

**Table 2.** Statistics for models characterizing cross-sectional and longitudinal associations between hippocampal structure and AD pathology

	Tau PET						Amyloid PET					
	Tau (main effect)			Tau * Time			Amyloid (main effect)			Amyloid * Time		
	*t*-value	*P*-value	df	*t*-value	*P*-value	df	*t*-value	*P*-value	df	*t*-value	*P*-value	df
R1	**−3.23**	**0.001**	**210**	1.15	0.252	1870	−1.32	0.189	208	0.79	0.429	1850
MTsat	−0.76	0.446	218	**−3.87**	**0.000**	**1890**	−0.51	0.608	218	−1.21	0.228	1870
R2*	−0.15	0.882	214	**−3.10**	**0.002**	**1880**	0.74	0.459	214	**−2.97**	**0.003**	**1860**
PD	0.93	0.352	192	**−4.24**	**0.000**	**1810**	0.84	0.401	193	**−3.03**	**0.003**	**1790**
Thickness	0.15	0.878	237	0.53	0.598	1920	−0.48	0.631	238	−0.52	0.605	1890

The results for the whole hippocampal models are shown here with component-specific effects in *SI Appendix*, Table S3. *P* < 0.05 effects are shown in bold. *P*-values calculated from robust model t-values and standard model degrees of freedom (df) (*Materials and Methods*).

Greater tau load was associated with lower hippocampal R1 at baseline which was statistically significant within all components. Tau load was not associated with any other microstructural measure, or hippocampal thickness at baseline. Greater tau load was associated with a more negative slope (steeper decline) in MTsat, R2*, and PD within the hippocampus over time. This effect was significant across all components in all cases except for R2* in component 3.

No associations were observed between amyloid index and hippocampal structure at baseline across the whole hippocampus. We did observe a significant negative association between amyloid load and hippocampal thickness specific to component 2 but it did not survive correction for multiple comparisons. Greater amyloid deposition was associated with more negative slopes of R2* (in whole hippocampus and in component 1) and PD (in whole hippocampus and in component 2).

No associations were observed between these measures of AD pathology and precentral gyrus structure, an a priori selected region of no interest (*SI Appendix*, Table S7).

### Hippocampal Microstructure Varies by APOE Genotype.

The APOE gene, specifically the APOE4 variant, confers significantly increased risk for AD (46). We examined hippocampal structural differences between APOE4 carriers and noncarriers. One subject was missing genotype data, so we examined APOE4 carrier differences in the other 223 older individuals Fig. 3). As with previous analyses, we tested whether associations were present at baseline, and whether longitudinal change in structure was associated with APOE4 carrier status, correcting for age, sex, and years of education. Associations were tested across the whole hippocampus and within individual spatial components. Statistics for these models are shown in Table 3 (whole hippocampus statistics) and *SI Appendix*, Table S4 (including component-specific statistics).

**Table 3.** Statistics for models characterizing cross-sectional and longitudinal associations between hippocampal structure and APOE4 carrier status

	APOE4					
	APOE4 (main effect)			APOE4 * Time		
	*t*-value	*P*-value	df	*t*-value	*P*-value	df
R1	**−2.85**	**0.005**	**241**	**3.27**	**0.001**	**2070**
MTsat	−0.49	0.622	249	**−2.69**	**0.007**	**2090**
R2*	**−2.67**	**0.008**	**249**	1.16	0.245	2090
PD	−0.28	0.780	226	−1.07	0.283	2020
Thickness	−1.31	0.191	272	−0.34	0.734	2140

The results for the whole hippocampal models are shown here with component-specific effects in *SI Appendix*, Table S4. Effects where *P* < 0.05 are shown in bold. *P*-values were calculated from robust model *t*-values and standard model degrees of freedom (df).

In APOE4 carriers (n = 89), we observed significantly lower hippocampal R1 and R2* at baseline compared to noncarriers (n = 134) which was significant across all components for both measures. No APOE4 group difference was observed for whole hippocampal thickness, but carriers did have lower thickness localized to component 2 compared to noncarriers. R1 decreased faster over the follow-up time in noncarriers than in carriers across the whole hippocampus, driven by component 2 (the component 1 effect did not survive multiple comparisons correction). Conversely, MTsat decreased faster in carriers, in the whole hippocampus, driven by component 1.

### Hippocampal Structure Relates to Cognition in People With More Pathology.

**Fig. 4.**
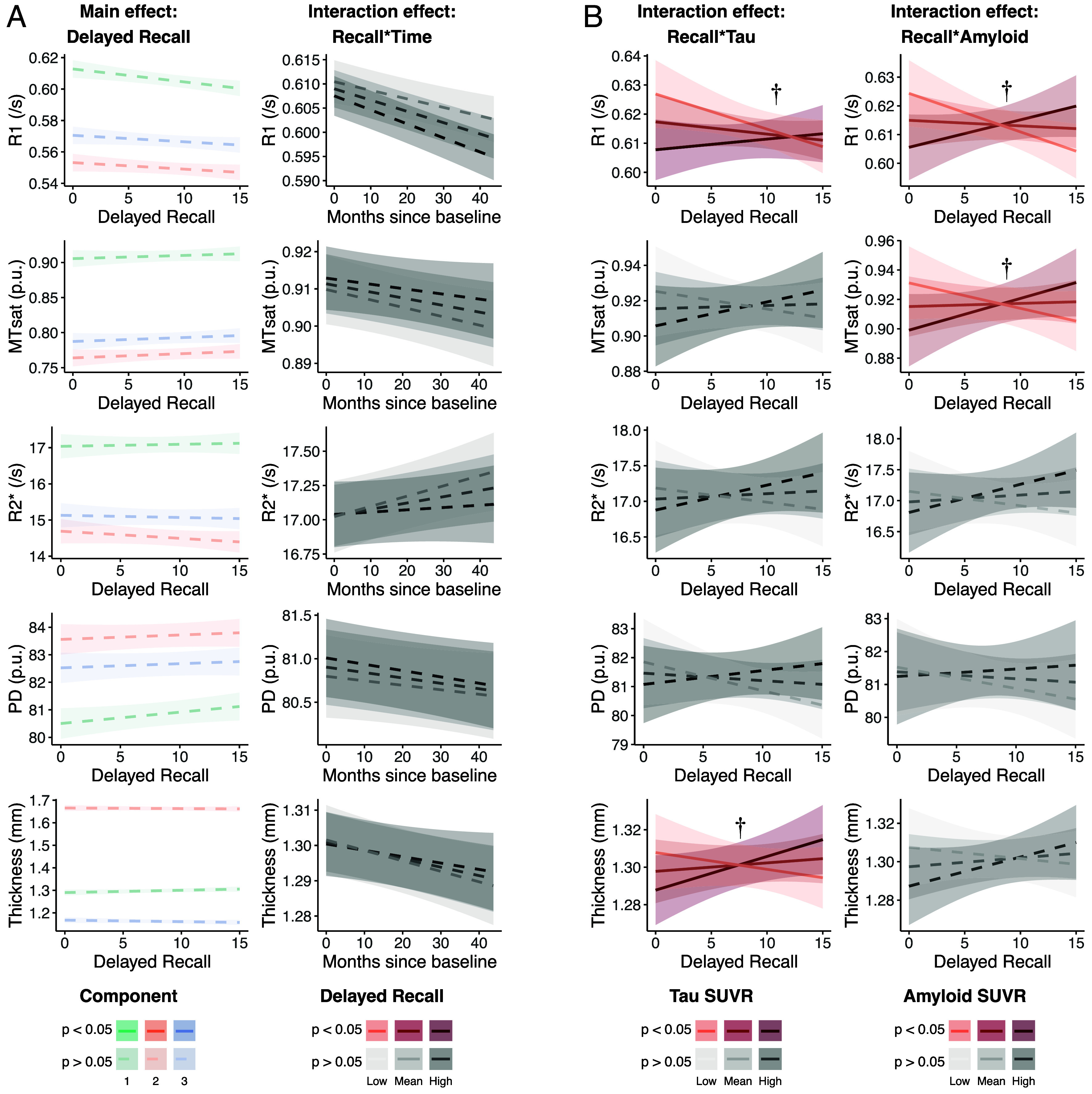
Characterizing cross-sectional and longitudinal associations between hippocampal structure and delayed recall ability. Each row corresponds to a structural measure. Each column represents a main effect from the mixed-effects models. (*A*) shows the main cross-sectional effects (*Left* column) and the interaction between the main effect and time (*Right* column). (*B*) shows the interaction between delayed recall and AD pathology (*Left* column: Tau, *Right* column: Amyloid), as it relates to structure. Faded and dotted plots represent nonsignificant effects. For clarity, longitudinal effects are not shown for each component but represent the whole hippocampal model results. Data within each plot are partial-residuals, meaning they are corrected for other variables in the model. In SUVR legends, Low and High indicate 1 SD below/above the mean, respectively. Effects marked with a dagger (†) were not significant after correcting for multiple comparisons. For clarity, regression plots are shown as mean ± SE summary lines. Full data distributions are shown in *SI Appendix*, Fig. S2. Semiquantitative measures (MTsat and PD) are expressed in percentage units (p.u.).

Hippocampal damage has been shown to relate to age- and Alzheimer’s-related episodic memory impairment, in particular tests of delayed recall (58–64). We tested the associations between hippocampal macro- and microstructure and delayed recall score on the Rey Auditory Verbal Learning Task (RAVLT), correcting for age, sex, and years of education (Fig. 4). Full statistics for these models are shown in Table 4 (whole hippocampus statistics) and *SI Appendix*, Table S5 (including component-specific statistics).

**Table 4.** Statistics for models characterizing cross-sectional and longitudinal associations between hippocampal structure and delayed recall ability

	DR model (longitudinal)						DR * pathology models (baseline)					
	DR (main effect)			DR * Time			DR * Tau			DR * Amyloid		
	*t*	*P*	df	*t*	*P*	df	*t*	*P*	df	*t*	*P*	df
R1	**−2.12**	†**0.034**	**1670**	−1.24	0.213	2130	**2.23**	†**0.027**	**181**	**2.72**	†**0.007**	**181**
MTsat	0.98	0.329	1440	−0.03	0.977	2080	1.28	0.202	181	**2.22**	†**0.028**	**181**
R2*	0.17	0.869	1480	−1.23	0.218	2090	0.92	0.358	181	1.55	0.124	181
PD	1.51	0.132	2080	0.20	0.844	2130	1.31	0.193	181	0.86	0.392	181
Thickness	-0.18	0.858	1150	0.58	0.559	1950	**2.02**	†**0.045**	**180**	1.32	0.190	180

The results for the whole hippocampal models are shown here with component-specific effects in *SI Appendix*, Table S5. Effects where *P* < 0.05 are shown in bold, however no effects survived correction for multiple comparisons within each test (column) (marked with a dagger, †). *P*-values were calculated from robust model *t* -values and standard model degrees of freedom (df).

The primary analysis (n = 218) revealed a negative association between R1 and delayed recall in the baseline whole hippocampal analysis, however this did not survive correction for multiple comparisons. No other significant effects were seen in other components or with other structural measures. We did not observe any interaction between change in structure over time and delayed recall in the whole hippocampus. R1 in component 1 declined faster over time in people with poorer delayed recall scores, an effect that did not survive multiple comparisons correction.

We conducted a post hoc analysis on subjects with PET data (n = 188) to explore whether a stronger relationship between structure and cognition was present at baseline in people with greater AD pathology. We do observe a significant direct association between worse delayed recall and greater tau load [t(295) = −2.07, *P* = 0.040] but not greater amyloid [t(295) = −1.52, *P* = 0.130].

We observed a positive interaction of tau load and delayed recall on R1, with greater R1 being associated with better recall in people with more tau, however this was not statistically significant after correcting for multiple comparisons. This effect was driven primarily by component 3. A similar interaction was seen with hippocampal thickness, whereby greater thickness was associated with better recall in people with more tau, an effect that was driven by component 2. The same was not seen for other measures of hippocampal microstructure.

A positive interaction of amyloid load and delayed recall was also found on R1 (across all components) and MTsat (driven primarily by component 2) that also did not survive correction for multiple comparisons. This effect was not observed for R2*, PD, or thickness.

## Discussion

In this study, we demonstrate the utility of in vivo histology through subregional and multimodal qMRI parameterization to provide greater biological interpretability than typically used macrostructural measures, opening a window to understanding neuropathological mechanisms in the earliest stages of age- and disease-related neurodegeneration. We topographically mapped measures of microstructure and macrostructure across the unfolded surface of the hippocampus, describing three spatial regions of unique structural covariance between four microstructural parameters (R1, MTsat, R2*, PD), and macrostructure (allocortical surface thickness). We demonstrate both cross-sectional and longitudinal associations of these measures with healthy aging across the lifespan with subtly varying effects across different spatial areas of the hippocampus. Critically, we show significant associations of AD pathology and APOE4 carrier status with microstructural, but not macrostructural measures. Furthermore, we show some evidence of associations between delayed recall ability and hippocampal structure in individuals with higher levels of AD pathology, though these results did not survive multiple comparisons correction. Our findings support demyelination and increased iron broadly across the hippocampus as key hallmarks of the aging hippocampus. We also provide evidence that a combination of microstructural changes (as opposed to any singular factor) occur prior to macroscopic atrophy and that such microstructural changes map more closely to tau levels than amyloid.

### Spatial Segmentation of the Hippocampus.

We provide evidence for three distinct topographical regions across the hippocampus defined by our structural measures. In all components, we saw patterns of R1, MTsat, and R2* positively covarying with each other and negatively covarying with PD. This supports a model in which hippocampal microstructure is primarily defined, according to these microstructural measures, by macromolecular content, likely driven by myelin (65, 66). Component 1 highlights thinner but highly myelinated areas corresponding to posterior subiculum/CA1. Component 2 encompasses thicker but minimally myelinated regions corresponding to the hippocampal head. Finally, component 3 defines another thinner area but with relatively lower myelination than component 1 in an area corresponding largely to posterior CA2/3.

These maps, in particular that of MTsat which is the strongest correlate of myelin of all measures studied (25), have high correspondence with previously aggregated ex vivo measures of R1 at 9.4 T, and in vivo MT and T1 relaxation maps obtained at 7 Tesla (67), as well as previously reported T1w/T2w maps (7, 45) (a commonly used proxy for myelin in lieu of quantitative sequences (68)). High values for MRI-derived myelin measures have previously been observed along the length of the subiculum (6, 7, 45, 68). Histological studies also report the presence of high myelination in this region from fibers of the perforant path and cingulum bundle (35, 69, 70). In contrast, lower myelination was seen in the other components, likely reflecting the sparser pyramidal cell organization structure and unmyelinated mossy fibers and schaffer collaterals projecting within and between CA regions (69).

Our reported segmentation is also highly aligned with a previous T1-weighting-based clustering of hippocampal voxels (71). In that study, open-access datasets were leveraged to compare structural clustering across the hippocampus within individuals at different stages of life and neurodegenerative disease, revealing three regions of structural covariance, respectively within the hippocampal head, subiculum, and body/tail CA regions. The correspondence of our findings to those previously published (71) support the generalizability of our segmentation between mapping methods (surface vertices vs. voxels) and clustering techniques (OPNMF vs k-means clustering). Differences with other studies which found either four- (6) or six-component solutions (7) can likely be attributed to differences in the features used to define structure (e.g. inclusion of measures of diffusion).

### Hippocampal Macrostructure and Microstructure in Healthy Aging.

We demonstrate both cross-sectional and longitudinal associations of hippocampal structure with healthy aging across the lifespan. Associations between age and microstructure were almost exclusively detected in the older age group, whereby R1, MTsat, and R2* were all negatively correlated with age, indicative of a loss of macromolecular tissue content, possibly driven by demyelination (26). Demyelination is unlikely to be the only change occurring over time however, as steep longitudinal decreases in R1 were observed within the older adult group across all spatial components, whereas decreases in MTsat were localized only to the subiculum/CA1 subregion. Due to its broader range of sensitivities compared to other measures, R1 may be better equipped to detect subtle, simultaneous changes in multiple biophysical environmental factors associated with aging, such as water motility, inflammation, atrophy, and iron deposition (23, 31).

Our data support previous findings on nonlinear lifespan trajectories of brain microstructure (72–77). The nonlinear trajectory is particularly apparent for R2*, which was higher in older compared to younger adults and indeed increased longitudinally across the hippocampus but showed a localized negative cross-sectional association with age within older adults. Although a strong linear mapping between iron and R2* is observed in low-myelin deep gray matter structures (24, 78), R2* within the hippocampus is influenced both by myelin (28, 79) and iron deposits (80, 81). Though R2* was seen to be higher in older adults than younger adults, our data do not suggest a lifetime increase in myelination as no age-group difference in R1 and MTsat was observed. Rather, the age-group R2* difference is more likely due to increased iron in older adults. The negative association between age and R2* in component 3 (posterior CA2/3) in older adults is in line with that of R1 and MTsat, indicating a loss of macromolecular tissue content, which may suggest a driving force of age-associated demyelination. This effect apparently outweighs any opposing effect of further increased iron within this subregion, though the effect was small as might be expected in a situation with such conflicting driving factors. The specificity of this effect to component 3 suggests either that age-related loss of macromolecular content (possibly indicating demyelination) is specific to this region, or that it is similar across all components but the confounding effect of increased iron as a function of age is weaker in this region relative to components 1 and 2. In support of the latter point, the age group difference was not observed within component 3, suggesting a lesser influence of iron within this subregion. These changes demonstrate the complex nature of interpreting changes in R2* in this context and at different life stages, as different elements of the biophysical environment can have opposing effects, canceling each other out and masking subtle changes (24, 65). Disentangling paramagnetic and diamagnetic susceptibility effects will be essential to fully understand early trajectories of these factors.

We also observed nonlinear patterns of macrostructural change across the lifespan. A subtle cross-sectional positive association between hippocampal head thickness and age in younger adults and thinner hippocampal head thickness in older adults compared to younger adults were observed but did not survive correction for multiple comparisons. However, similar to the effects of R1 and MTsat, robust negative associations with cross-sectional age and longitudinal decline were observed for thickness. Lower thickness in older age is expected given age-related atrophy (10, 82–84). In contrast, the positive relationship with age in younger adults may reflect later stages of hippocampal development. Some models seem to support hippocampal growth into the third decade of life (85, 86), while other studies suggest gray matter stops increasing around or before age 20, though few report hippocampal-specific effects, and instead focus more broadly on whole-brain gray matter volume or cortical thickness (77, 87–89).

An unexpected observation of our study was the longitudinal decrease in hippocampal PD in older adults, indicative of decreases in water content over time. This is in contrast to most reports of PD in older age and disease whereby inflammation and subtle atrophy drive increases in PD (29, 30, 73). Cross-sectional associations between older age and lower PD in subcortical gray matter have been noted previously (90), where it was attributed to residual T2*-weighting of the apparent PD signal which caused age-related increases in iron to decrease the PD signal. This explanation does not hold for our study as we have corrected for the multiexponential T2* signal decay through extrapolation of the signal decay in PDw images to TE = 0 (21). We also do not believe this is an artifact of systematic partial voluming from high-PD white matter, as we do not see the increases in MTsat that would be expected in this case. Another explanation for the previous finding is that PD signal was calibrated to a fixed value within white matter of 69 p.u. The recommendation of this benchmarking technique was validated for use in young brains free from pathology (29), and does not account for changes in white matter composition that occur in age and disease, which introduces a confound. For this reason, we took an alternative approach of fixing PD values relative to a CSF value of 100 p.u. (91), as CSF water content is not known to change as a function of age or disease. This may explain why we do not see a cross-sectional association between older age and lower PD, however the longitudinal effect is apparent. The remaining effect is unlikely to be an artifact of the normalization, as longitudinal PD decline is not seen in another subcortical gray matter comparison region, the thalamus (Supporting Information). PD may here be reflecting shifts in different water compartments, for example intra- vs. extracellular vs. intramyelin water, or complex mechanisms such as recruitment of nonneuronal cells via processes such as astrocytosis (92–95). Multishell diffusion imaging could provide valuable insight into the biophysical mechanisms at play here, however, higher-than-typical resolutions would be required to accurately interpret diffusion changes across different regions of the hippocampal surface (96, 97).

### Hippocampal Microstructure, But Not Macrostructure, Relates to AD Pathology.

A critical observation from this study is that greater Alzheimer’s-localized tauopathy and amyloid burden were related to measures of hippocampal microstructure but were unrelated to hippocampal macrostructure (one association between amyloid and macrostructure was observed but did not survive correction for multiple comparisons). These findings underscore the value of qMRI in measuring tissue microstructure and providing biologically interpretable measures of pathology. Previous studies have shown that MRI-derived measures of microstructure from quantitative sequences or radiomics provide distinct information to macrostructural measures like volume (98–100, 23, 101). Such measures can aid detection of pathological but therapeutically rescuable tissue in incipient AD by revealing alterations in tissue biophysical environment prior to macroscopic atrophy, after which point any treatments that slow or delay disease progression trajectories may have poor efficacy in preventing cognitive decline (102, 103).

Our data also suggest that R1 may be sensitive to tauopathy at an earlier stage than any other measure that we tested. At baseline, we observed that individuals with greater tauopathy had significantly reduced R1 compared to those with lower tauopathy. In contrast, MTsat, R2*, and PD appear similar at baseline but diverge over the follow-up period to the point where, at later timepoints, a significant difference is apparent across different levels of pathology. R1 changing earlier is also supported by the APOE4 analysis, whereby R1 is lower in carriers than noncarriers at baseline, but over time noncarriers decline faster and “catch up” to carriers. In contrast, there is no reliable difference in hippocampal MTsat at baseline, but MTsat does decline faster over time in carriers than noncarriers. R2* was also lower in carriers at baseline than noncarriers, likely driven by complex alterations in the balance of iron and myelin in early stages of pathology.

Greater sensitivity of a given measure to pathology could also be attributed to greater measurement reliability across scans. We could not fully assess test–retest reliability of MPM measurements in our cohort however a previous study showed low within-subject variability in gray matter in a cohort of young adults (104). It was shown that R1 (2.9%) and especially R2* (4.1%) had significantly higher within-subject variability compared to MTsat (2.3%) and PD (1.9%). Furthermore, comparing left and right hemispheres as an approximation of test–retest reliability, we observed comparable reliability of R1 and MTsat, which each have greater estimated reliability than R2*. A particularly high estimated reliability of PD was also observed (Supporting Information: Reliability Analyses, *SI Appendix*, Fig. S4). With these data in mind, our observed finding of greater sensitivity of R1 is unlikely attributable to lower measurement variability relative to other maps, though future studies should consider scanning a test–retest subset where possible.

### Hippocampal Structure Relates to Cognition More Strongly in Incipient AD.

The only univariate relationship between delayed recall performance and hippocampal structure that we observed was a negative correlation between R1 and delayed recall, although this effect did not survive correction for multiple comparisons and should therefore be interpreted with caution. Despite this limitation, this trend was surprising given our previous analyses showing that lower R1 was associated with age and AD pathology. However, with increasing levels of pathology, the direction of this correlation becomes more positive, in line with our expectations. Said another way, our (tentative) findings suggest that people with AD pathology only perform well on a test of delayed recall if they have “healthy” hippocampal structure (greater R1, MTsat, or thickness).

Our findings are consistent with previous studies showing little to no structure–cognition relationship in healthy older adults (105–107) despite frequently reported associations between hippocampal structure and cognition in people with AD or mild cognitive impairment (58–64). It is possible that in early stages of degeneration, despite significant alterations detected by qMRI measures, cognitive compensation mechanisms may obscure straightforward associations between reduced structural integrity of brain tissue and cognitive performance (108). Our tentative finding suggests that even in people who are cognitively normal but who have the earliest signs of AD pathology, memory performance becomes increasingly dependent on or bottlenecked by hippocampal integrity. This effect was observed when AD pathology was quantified by either amyloid or tau and therefore appears to be a general feature of AD risk, rather than, for example, exclusively related to tau-specific neurotoxicity. These results are complemented by our supplemental analysis where we showed that hippocampal qMRI metrics were unable to explain significantly more variance in delayed recall performance over and above that provided by global PET measures. It is clear that the variation in cognitive performance in both normal aging and incipient AD is largely driven by factors other than those our qMRI metrics are sensitive to, or by regions other than (or in addition to) the hippocampus.

We emphasize great caution in interpreting any findings that did not survive our criteria for multiple comparisons correction. We report the data as we observe it and offer some interpretation as to their potential meaning, but we await replication in independent datasets when possible to confirm the robustness of these findings.

### Conclusions.

In this paper, we have shown that in vivo measures sensitive to tissue microstructure can accurately capture known variance in microstructure across the hippocampus. Using these qMRI measures (or “in vivo histology”) we support demyelination and increased iron deposition as key hallmarks of the aging hippocampus. We provide evidence that a combination of microstructural changes occur prior to macroscopic atrophy in AD, not one feature in particular, indicated by particular sensitivity of R1 to tauopathy and APOE4 status. Furthermore, despite clearly distinct subregions and considerable variation in macro- and microstructural parameters between them, most effects with aging and AD-related factors were similar across subregions, with the magnitude of effects differing subtly in most cases. Finally, while we report tentative evidence for an association between cognition and hippocampal microstructure in incipient AD, our findings suggest that cognitive variation in aging and early AD is largely influenced by factors beyond hippocampal microstructure as captured by our qMRI metrics.

qMRI measures provide greater biological interpretability than macrostructural measures, opening a window to understanding neuropathological mechanisms in the earliest stages of age- and disease-related neurodegeneration. A combination of macrostructural and microstructural measures provides a more complete picture of brain health and disease, unlocking unique insights into the pathological state of brain tissue and the impact of AD at a point where therapeutic rescue of the tissue is most likely to be efficacious. Before translation to the clinic is possible, further work is required to fully elucidate parameter interpretation (e.g. of R2*) in cases where multiple biological processes may influence in different ways. Considerable progress has been made in standardizing analysis pipelines for qMRI but more work is required to further encourage adoption of a unified pipeline to calculate quantitative parameters (21, 109). Furthermore, the individual-level precision of these measures in patient and at-risk groups should be examined in greater detail to facilitate personalized treatment strategies. Ultimately, these measures promise to aid individualized diagnoses and improve prognoses for patients. Understanding the earliest hippocampal changes in both normal aging and AD are critical to better manage the increasing pressures of our aging population.

## Materials and Methods

We analyzed hippocampal microstructure in 261 cognitively healthy older adults with familial risk for AD using quantitative MRI (multiparametric mapping) and HippUnfold, a tool for unfolding the hippocampal CA mantle into a 2D plane. Orthogonal-projected nonnegative matrix factorization (OPNMF) was used to segment the hippocampal surface into three nonoverlapping regions based on covariance patterns in five structural measures (R1, MTsat, R2*, PD, thickness). PET imaging was used to assess tau and amyloid burden, and robust linear mixed effects models were employed to explore associations between hippocampal structure, pathology, APOE4 carrier status, and cognitive performance. Multiple comparisons were corrected using FDR, assuming a 10% FDR and an alpha level of 0.05. Full methodological information is detailed in *SI Appendix*, *Methods*.

## Supplementary Material

Appendix 01 (PDF)

Dataset S01 (DOCX)

## Data Availability

This study used data from the PResymptomatic EValuation of Experimental or Novel Treatments for AD (PREVENT-AD) study. Data availability for PREVENT-AD is governed by the Open Access protocols. Please refer to https://douglas.research.mcgill.ca/prevent-alzheimer-program/ for more information about PREVENT-AD. This study utilized a number of open access resources. Multiparametric maps were processed using hMRI toolbox (v0.5.0) (https://hmri-group.github.io/hMRI-toolbox/) in MATLAB. MRI images were denoised and registered using the openly available ANTs tools (110). Hippocampal surface unfolding and parameter mapping was conducted using the openly available HippUnfold toolbox (45) (https://github.com/khanlab/hippunfold). All data plots were organized and plotted using functions from the *tidyverse* R package (v2.0.0): https://github.com/tidyverse.
