# Supplementary material for: Quantitative MRI of the hippocampus reveals microstructural trajectories of aging and Alzheimer’s disease pathology
Source: Proc Natl Acad Sci U S A. 2025 Oct 27;122(44):e2502674122. doi: 10.1073/pnas.2502674122 (PMC12595451; doi:10.1073/pnas.2502674122)
Supplement: Supplementary file 1 — Appendix 01 (PDF) [file pnas.2502674122.sapp.pdf]

PNAS

Supporting Information for

Quantitative MRI of the hippocampus reveals microstructural trajectories of aging and Alzheimer's disease pathology

Alfie Wearn*, Christine L. Tardif, Ilana R. Leppert, Giulia Baracchini, Colleen Hughes, Jennifer Tremblay-Mercier, John Breitner, Judes Poirier, Sylvia Villeneuve, Boris C. Bernhardt, Gary R. Turner, R. Nathan Spreng* for the PREVENT-AD research group

Corresponding Author #1: Alfie Wearn
Email: alfie.wearn@mcgill.ca

Corresponding Author #2: R. Nathan Spreng
Email: nathan.spreng@mcgill.ca

This PDF file includes:

SI Methods

Supporting information: Determining optimal number of OPNMF components (Figure S1)
Supporting information: Within-component value means (and dispersion) (Table S1)
Supporting information: Longitudinal decreases in PD is not a global feature
Supporting information: Statistics for component-specific models (Tables S2-S5)
Supporting information: Sex differences between hippocampal qMRI parameters (Table S6)
Supporting information: Region of no interest does not relate to AD pathology (Table S7)
Supporting information: Hippocampal volume associations are in line with those of hippocampal thickness (Table S8)
Supporting information: Hippocampal qMRI does not explain more variance in delayed recall than global PET measures
Supporting information: Data Distributions (Figure S2)
Supporting information: Quality of hippocampal segmentation – example subjects (Figure S3)
Supporting information: Reliability analyses (Figure S4)
Supporting information: About PREVENT-AD
Supporting information: PREVENT-AD Research Group Authorship

SI References

Supporting Information: SI Methods

Participants

This study included 261 people from the ongoing PREVENT-AD study. 224 were older adults over the age of 60 with a first-degree familial history of AD. The remaining 37 were younger adults between the ages of 18 and 40. 142 older participants were followed up after 2-3 years.

All participants gave informed written consent before participating in the study. All procedures of the PREVENT-AD study were approved by the McGill institutional review board and/or the Comité d'éthique de la recherche du CIUSSS de l'ouest de l'ile de Montréal. The study was performed in accordance with the ethical standards of the 1964 Declaration of Helsinki.

Image acquisition

MRI scans were acquired on a 3T Siemens PrismaFit at the Douglas Research Centre, including a T1-weighted anatomical scan (Magnetization-Prepared Rapid Acquisition Gradient Echo (MPRAGE), 1mm isotropic resolution, TR/TE/TI=2300/2.96/900ms, FA=9°, TA=5:30), multi-parametric mapping (MPM)(1) and B1+ fieldmaps (described below). All sequences had whole-brain coverage.

We used MPM to measure R1 relaxation rate (1/T1), magnetization transfer saturation (MTsat), R2* relaxation rate and proton density (PD), each of which is differentially sensitive to different elements of tissue microstructure such as lipid, iron and water content. Three multi-echo gradient echo sequences were acquired (1mm isotropic resolution, TA=17:30) with predominant weighting for: T1 (TR=18ms, 6 echoes, TE=2.16-14.81ms, FA 20°), MT (TR=27ms, 6 echoes, TE=2.04-14.89ms, echo-spacing=2.57ms, FA 6°; MT pulse: 4ms Gaussian pulse, 2 kHz off-resonance, FA 220°) or PD (TR=27ms, 8 echoes, TE=2.04-22.20ms, echo-spacing=2.57ms, FA 6°). The MPM sequence has been shown to have good test-retest reliability at 3T(2).

$B1^+$ transmit field maps were acquired using two spin-echo echo-planar sequences with different flip angles (60°, 120°) and otherwise identical parameters (2x2x4mm resolution, TR/TE=4010/46 ms, TA=1:08).

Image processing

Microstructure maps (R1, MTsat, R2* and PD) were computed using hMRI toolbox v0.5.0 hMRI toolbox(3), including B1+ correction using the double angle method. RF receive sensitivity was estimated using the Unified Segmentation method. R2* maps were estimated using log-linear weighted least-squares ('WLS1').(4) By default, PD is calibrated by fixing white matter to an average of 69 p.u.. Given known age-related changes in white matter presenting a potential confound for this benchmark, we instead chose to calibrate PD to a CSF value of 100 p.u. as CSF water content is not known to change as a function of age or disease (5–7). The CSF calibration factor was calculated as the median apparent PD within voxels of the bilateral lateral ventricles for which T1 was > 3,800 ms (R1 < 1.26) in order to ensure conservative selection of 'true' CSF (8,7,9,10). For all other hMRI toolbox settings, default options were used. hMRI toolbox-produced measures of inter- and intra-contrast head movement were used for quality control. We excluded all subjects with any motion value outside the mean ± 3*IQR range.

MPM parameter maps and MPRAGE images were denoised using ANTS DenoiseImage(11). MPRAGE images were brain extracted by applying a brain mask calculated using Freesurfer

v7(12). The denoised, brain-extracted MPRAGE was then rigid registered to the denoised R1 map using antsRegistratonSynQuick.sh. The inverse transform was then applied to all MPM parameters maps to bring them all into MPRAGE space.

Hippocampal surface unfolding

HippUnfold is a recently developed programme that leverages the folded allocortical structure of the hippocampus, and unfolds it into a 2D plane(13). This procedure offers several advantages over typical hippocampal subfield masking methods, including: i) more accurate topological alignment between individuals despite individual-specific variation in folding structure, ii) greater respect for the topological continuity of subfields, iii) reduced partial volume effects due to mapping of parameters onto a 'mid-thickness' surface and iv) easier visualization of parameters across the entire hippocampal structure. Individualized masks of the grey matter and topological boundaries of each hippocampus of each subject were created using a 'U-net' deep convolutional neural network(14). After segmentation of the hippocampus using the T1w MPRAGE image, the allocortical strip from subiculum, through CA1, CA2, CA3 and CA4 is unfolded in a 2D midthickness surface with 7262 vertices, spaced 0.5mm apart. We used the HippUnfold output measure of 'thickness' as a marker of vertexwise macrostructure. MPRAGE-space MPM parameters were mapped onto surface space using 'wb_command - volume-to-surface-mapping' from Connectome Workbench(15). All five surface parameter maps (R1, MTsat, R2*, PD and thickness) were lightly smoothed with a Gaussian smoothing kernel (sigma =1mm) using 'wb_command -metric-smoothing' from Connectome Workbench.

HippUnfold produces a measure of overall masking quality by calculating a DICE metric compared with a whole hippocampus mask from FSL-FIRST. All those with value under 0.7 were manually inspected and excluded if errors in masking were identified. One session from each of five participants were excluded due to errors in hippocampal masking (two baseline, three follow-up sessions).

In this study we have analysed the hippocampal CA surface, including subiculum and excluding DG. We have excluded DG as it is not possible to acquire an accurate measure of 'thickness' on the resolutions and contrasts available, as it is a separate and much smaller cortical lamina. Furthermore, DG has been shown to be relatively resistant to age- and AD-related neuropathology and cell loss, with effects of interest most likely to exist within the CA mantle(16,17).

Analyzing hippocampal structure with HippUnfold specifically restricts insights to hippocampal gray matter. In order to maximise precision of assessing the topographical distribution of structural variables throughout the hippocampus, we have mapped values to a 'mid-thickness' surface – halfway between the external and internal (molecular layer) white matter areas. We cannot rule out the possibility of some partial voluming from these white matter areas, allowing us to pick up demyelination effects within e.g. the molecular layer of the hippocampus, particularly given the size of the hippocampal subfields relative to our 1mm^3 image resolution (18). However correspondence with previously published maps of the hippocampus produced at higher resolutions both *in vivo* and *ex vivo* (13,19,20) are encouraging as to the accuracy of our masking, and support our conclusions that effects are indeed taking place within voxels of the grey matter. A separate study focusing on the white matter layer would also be of great interest to fully understanding topographical distribution of structural changes in aging and AD. Studying the 'inner' surface rather than the 'mid-thickness' may enable this to be done using HippUnfold, but studies of white matter specifically would require higher resolution images to minimize partial voluming from grey matter voxels.

Orthogonal-projected non-negative matrix factorization

OPNMF(21,22) was used to segment the hippocampal surface into non-overlapping regions of covariance between the five structural measures, as has been done in previous studies(19,23). 'Orthogonally-projected' refers to the distillation of components that are non-overlapping and therefore represent distinct structural subregions defined by different covariance patterns in our structural parameters. The 'non-negative' constraint on inputs to OPNMF ensures outputs are

easier to interpret than alternative dimensionality reduction techniques such as principal component analysis. Using this method we create a group-level segmentation with boundaries derived from shared structure in the data across participants. This helps reduce inter-subject variability and enhances statistical power, as all subjects are represented in a common component space. Importantly, this aligns conceptually with traditional group-level parcellations but provides a data-driven alternative tailored to the specific features studied here (R1, MTsat, R2*, PD, thickness).

Briefly, OPNMF decomposes a non-negative input matrix X of dimensions $m \times n$ into two matrices: a non-negative component matrix W of dimensions $m \times k$ and a non-negative weight matrix H of dimenions $k \times n$ where k representes the *a priori* chosen number of components. The goal is to find the optimal W and H such that their product approximates X as closely as possible ($X \sim W \times H$), while enforcing orthogonality on W, calculated by minimizing a cost function. The orthogonality allows each hippocampal surface vertex to be assigned a single output component. Each structural parameter within a given hemisphere of each subject correspond to values in H.

In our case m represents the number of hippocampal midthickness vertices (7262) and n represents the number of structural features (5) multiplied by the number subjects with good quality baseline data (260) (total: 1300), with subjects nested within features. Left and right hemispheres were processed separately, and only baseline sessions were used to fit the model. Prior to calculating the OPNMF, the input data were winsorized at 5% to exclude any extremely outlying values for each hemisphere, structural parameter and subject. Input data were then z-scored within-feature and within-hemisphere (but between-subject and between-vertex), and then shifted by the minimum value from all z-scored metrics to ensure all values were >0.

Follow-up session data were winsorized identically to the baseline data. The z-score transform was calculated using the baseline data fit. The OPNMF fit from baseline data was also then applied to the follow-up data, to ensure comparability of the identified components and loadings.

The OPNMF was implemented using the python package '*opnmf*' (v 0.0.2). The ability to run an 'out of sample' transform of the OPNMF was not implemented in the '*opnmf*' package, so an amendment was made and is available on Github: https://github.com/Alfiew/opnmf. In this case, W (definition of which vertex belongs to which component) was kept stable and H (loadings of subject features) was recalculated given a new X matrix.

The optimal number of components is determined through a stability analysis, following the same procedure as previous studies (19,23). Briefly, the baseline dataset was split into two groups of equal size, and OPNMF was run on each split independently. The cosine similarity of the component matrix W was calculated within each split. Finally, the product-moment correlation of the cosine similarities between the splits was calculated. This gave an r-value for each vertex of the hippocampus that indicates the similarity of the two runs. The mean r-value across all vertices was the final stability metric (representing similarity of spatial components across random splits). This was repeated eight times for each level of k from 2 to 7. We also assessed the accuracy of each reconstruction, for each of the eight repeats. This was measured using the gradient between successive increases in k in the mean squared error of the OPNMF reconstruction of X(decline in reconstruction error with each additional component). This is predicated on the idea that each additional level of k can be expected to decrease the mean square error given a more comprehensive model, but each additional addition may result in decreasing benefit compared to the preceding level.

Stability was very high (>0.9) for 2- and 3-component solutions, after which further components carry a higher cost in reconstruction stability, or, in the case of the left-hemisphere 4-component solution, greater frequency of convergence failures. The gradient of reconstruction error change between 2- and 3-component solutions is strongly negative, and rapidly becomes flatter with each additional component. We therefore concluded that a 3-component solution was optimal using our chosen parameters. Left and right hemispheres showed similar stability and accuracy at most

stages. Convergence failures were increasingly seen with ≥4-component solutions. Results are shown in Fig S1.

Positron Emission Tomography

PET data was collected and processed as described in a previous publication (24).

Brain tau was assessed using [18F] Flortaucipir PET imaging in a 'meta ROI', an average of Standardized uptake value ratio (SUVR; relative to inferior cerebellar grey matter) in brain regions known to be affected by tau early in AD, including entorhinal cortex, parahippocampal, inferior temporal, middle temporal, and fusiform gyri, and the amygdalae(25).

Similarly, brain amyloid was assessed using [18F]NAV4694 PET imaging, using the amyloid index, a global summary metric of amyloid load in the cerebral cortex. It is an average of the SUVR (relative to cerebellar grey matter) within the following bilateral regions from the Desikan-Killany atlas(26): caudalmiddlefrontal, lateralorbitofrontal, medialorbitofrontal, parsopercularis, parsorbitalis, parstriangularis, rostralmiddlefrontal, superiorfrontal, frontalpole, inferiorparietal, precuneus, superiorparietal, supramarginal, caudalanteriorcingulate, posteriorcingulate, isthmuscingulate, rostralanteriorcingulate, middletemporal, superiortemporal, inferiortemporal.

PET imaging was not necessarily concurrent with MRI. PET scans were conducted on average 299±709 days prior to MRI collection

Values of tau and amyloid SUVR were greatly right skewed, so an inverse reciprocal transform was performed prior to further analysis to reduce disproportionate influence of extreme values on findings. These distributions are shown in Fig S2

Statistical Analysis

To describe hippocampal structural changes over the lifespan, we ran a series of robust linear mixed effects models whereby the loading of each of the five structural parameters (with a separate model per parameter) was predicted by age. Two models were run for each structural measure: 1) to examine overall hippocampal effects; 2) to explore spatial component-specific effects.

Within the age models we tested groupwise cross-sectional age differences (older vs younger) as well as within-group associations between baseline age (age_*bl*) and structure. To examine longitudinal change over time, we also included a parameter for time since baseline. We also included sex as a covariate of interest. Results for the main effect of sex are shown in Supplementary Material (S. Table 1). For both models we accounted for differences in structure between hemispheres and components. For component-specific models we allowed all age terms (and sex) to vary independently across components. To account for within-subject correlation of outcome measures over time and within components and hemispheres, we included subject ID as a random intercept. The final model structures were as follows, with main effects of primary interest shown in bold:

Whole hippocampal age model:
*Structure ~ hemisphere + component + (**age_group + age_group:age_bl + time_since_baseline** + sex) + (1 | subject)*

Component-specific age model:
*Structure ~ hemisphere + component + **component:(age group + age group : age at baseline + time since baseline** + sex) + (1 | subject)*

Similar model structures were used to assess the cross-sectional and longitudinal associations between structure and 1) AD pathology (tau and amyloid burden), 2) APOE4 carrier status and 3) Delayed Recall, both across the whole hippocampus and across individual spatial components. Age, sex and years of education were included as covariates, and a random intercept for each subject was included. Models are described below, with 'PET' representing tau or amyloid SUVR, APOE4 carrier status or RAVLT delayed recall.

Whole hippocampal model:
Structure ~ hemisphere + component + ***PET * time since baseline*** *+ age at baseline + sex + education + (1 | subject)*

Component-specific model:
Structure ~ hemisphere + component + ***component:(PET * time since baseline)*** *+ age at baseline + sex + education + (1 | subject)*

These models were also run on the structural measures of an *a priori* selected region of no interest, precentral gyrus, to infer whether hippocampal structure associations with pathology were a common 'whole brain' feature. Results for this analysis are shown in Supplemental Information (S. Table 2).

Finally, to explore structure-cognition relationships across differing levels of pathology, we created additional models, identical to those above but 1) with an interaction term for tau or amyloid PET and 2) examining cross-sectional data only, therefore without an interaction term for longitudinal time:

Whole hippocampal model:
Structure ~ hemisphere + component + ***delayed recall * PET*** *+ age at baseline + sex + education + (1 | subject)*

Component-specific model:
Structure ~ hemisphere + component + ***component:(delayed recall * PET)*** *+ age at baseline + sex + education + (1 | subject)*

For all models with hippocampal thickness as the structural measure, intracranial volume was added as an additional covariate to account for greater hippocampi due to larger head size.

All models were also run with hippocampal volume as a dependent variable to enable easier comparison to the literature. Results for these analyses are shown in S. Table 3.

P-values for the robust mixed effects models were estimated from degrees of freedom calculated on the non-robust models using the Satterthwaite method, and the t-values from the robust models.

All models were run in R v4.3.1. Robust mixed effects models were run using the '*robustlmm*' R package v3.3-1(27). Non-robust mixed effects models (for estimation of degrees of freedom) were run using lme4 v1.1-35.2(28).

Within this study we have performed a large number of statistical tests, thus increasing the risk of false positive findings. To address this, we have corrected our statistical tests for multiple comparisons using the false discovery rate (FDR) calculation, assuming a 10% FDR and an alpha level of 0.05. FDR correction was performed separately for each main effect of the models, as each addresses a different hypothesis. Within each main effect, we therefore corrected across 20 values: four models (one whole hippocampal model plus three component-specific models) in each of five structural parameters. This method provides a balance between minimizing false positive and maximizing true positive findings. Raw, uncorrected p-values are shown but effects that no longer reach criteria for statistical significance are indicated (†) within the data tables. Wherever possible we have employed robust statistical techniques, resistant to outlying datapoints, and constructed comprehensive regression models to precisely and accurately describe patterns within the data. Our findings will provide a valuable reference to future studies with independent datasets which would serve to confirm or deny the robustness of our findings.

Supporting Information: Determining optimal number of OPNMF components

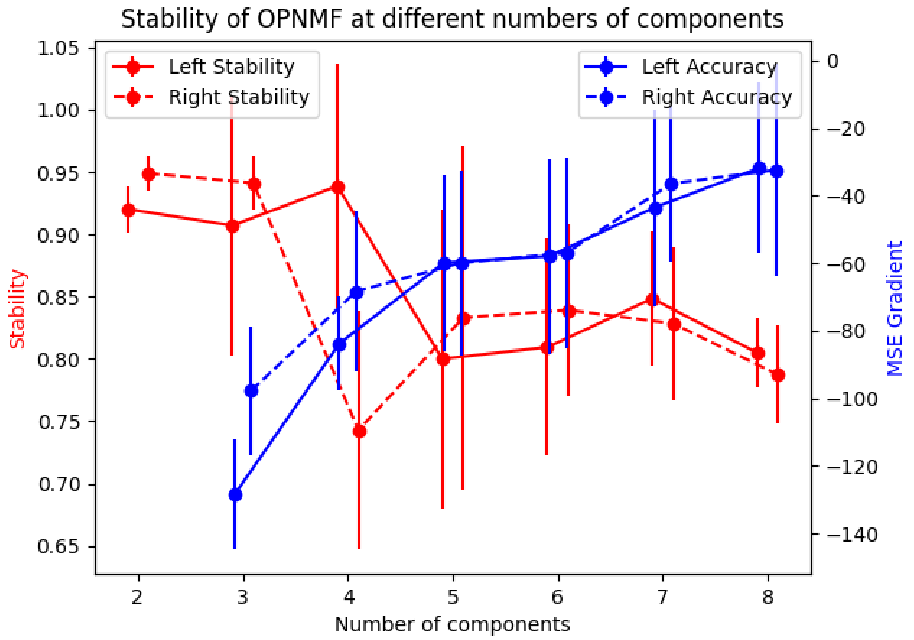

Figure S1. Stability and accuracy for varying numbers of OPNMF component solutions. The MSE (mean squared error) gradient at each point represents the change in MSE between that number of components and the solution with one fewer component. Points represent mean ± standard deviation across 10 repeats. Points are jittered slightly to allow visualisation of error bars. Stability was very high (>0.9) for 2- and 3-component solutions, after which further components carry a higher cost in reconstruction stability, or, in the case of the left-hemisphere 4-component solution, greater frequency of convergence failures. The gradient of reconstruction error change between 2- and 3-component solutions is strongly negative and rapidly becomes flatter with each additional component. We therefore concluded that a 3-component solution was optimal using our chosen parameters.

Supporting Information: Within-component value means and regional dispersion

Table S1 | Component values and within-subject regional dispersion for each structural measure

		Mean value (SD)	Mean dispersion (SD)
R1 (/s)	Whole Hipp.	0.574 (0.037)	0.034 (0.010)
	Component 1	0.607 (0.031)	0.036 (0.011)
	Component 2	0.548 (0.025)	0.031 (0.009)
	Component 3	0.568 (0.026)	0.033 (0.009)
MTsat (p.u.)	Whole Hipp.	0.822 (0.085)	0.081 (0.023)
	Component 1	0.910 (0.067)	0.090 (0.024)
	Component 2	0.766 (0.052)	0.075 (0.022)
	Component 3	0.791 (0.053)	0.076 (0.021)
R2* (/s)	Whole Hipp.	15.542 (1.824)	2.073 (0.821)
	Component 1	17.033 (1.617)	2.232 (0.868)
	Component 2	14.498 (1.437)	2.060 (0.852)
	Component 3	15.096 (1.341)	1.926 (0.707)
PD (p.u.)	Whole Hipp.	82.128 (2.916)	2.025 (0.692)
	Component 1	80.579 (2.650)	2.099 (0.666)
	Component 2	83.398 (2.714)	2.188 (0.745)
	Component 3	82.407 (2.656)	1.789 (0.593)
Thickness (mm)	Whole Hipp.	1.367 (0.224)	0.269 (0.168)
	Component 1	1.293 (0.064)	0.176 (0.032)
	Component 2	1.656 (0.093)	0.495 (0.073)
	Component 3	1.153 (0.052)	0.136 (0.029)

Supporting information: Longitudinal decreases in PD are not a global feature

In order to examine if the unexpected longitudinal decrease of PD was an artefact of normalization, we looked at a different subcortical grey matter region, the thalamus, to see if the same effect was observed. We extracted PD values from the thalamus using a mask created using FreeSurfer. We did not see the same pattern in the thalamus of PD increase over time, correcting for age, sex and education ($t(177)=-1.01$, $p=0.312$), indicating at least some specificity of the effect to the hippocampus. A whole-brain study of these metrics is beyond the scope of the current study but would be of interest.

Supporting information: Statistics for component-specific models

Table S2 | Statistics for models characterizing cross-sectional and longitudinal lifespan effects of hippocampal macro- and micro-structure. For each structural measure, a model was run without exploring component-specific interactions ('Whole Hipp.' Model), for maximum statistical power in detecting non-spatially-specific effects. Then, to characterize such spatial effects, a model was run in which the effects were allowed to vary for each component. The results for each component and for the whole hippocampal model shown in each row. Effects where p<0.05 are shown in bold, with a dagger (†) indicating effects that do not survive correction for multiple comparisons. P-values were calculated from robust model t-values and standard model degrees of freedom (df).

		Age Group			Age (Young)			Age (Older)			Longitudinal change		
		t-value	p-value	df	t-value	p-value	df	t-value	p-value	df	t-value	p-value	df
R1	Whole Hipp.	0.67	0.501	278	1.21	0.228	278	**-6.64**	**0.000**	**253**	**-7.55**	**0.000**	**2270**
	Component 1	1.04	0.297	481	1.65	0.100	481	**-5.37**	**0.000**	**377**	**-5.82**	**0.000**	**2190**
	Component 2	1.07	0.287	481	0.75	0.457	481	**-5.82**	**0.000**	**377**	**-3.30**	**0.001**	**2190**
	Component 3	-0.37	0.715	481	0.69	0.489	481	**-6.68**	**0.000**	**377**	**-4.82**	**0.000**	**2190**
MTsat	Whole Hipp.	-1.42	0.157	287	0.76	0.450	287	**-5.13**	**0.000**	**254**	**-3.25**	**0.001**	**2300**
	Component 1	-1.32	0.186	555	0.77	0.443	555	**-4.14**	**0.000**	**415**	**-3.99**	**0.000**	**2200**
	Component 2	-0.44	0.658	555	0.58	0.565	555	**-4.47**	**0.000**	**415**	-1.02	0.308	2200
	Component 3	-1.88	0.061	555	0.69	0.488	555	**-4.99**	**0.000**	**415**	-1.11	0.265	2200
R2*	Whole Hipp.	**3.45**	**0.001**	**292**	1.39	0.167	292	-1.24	0.216	257	**2.21**	**0.027**	**2300**
	Component 1	**4.41**	**0.000**	**571**	1.06	0.291	571	-0.29	0.772	425	1.70	0.088	2210
	Component 2	**2.82**	**0.005**	**571**	1.26	0.209	571	-0.71	0.478	425	1.59	0.113	2210
	Component 3	1.61	0.108	571	1.19	0.235	571	**-2.27**	**0.024**	**425**	0.89	0.375	2210
PD	Whole Hipp.	0.72	0.472	266	0.23	0.821	266	0.06	0.953	254	**-3.25**	**0.001**	**2220**
	Component 1	0.69	0.489	357	-0.20	0.843	357	-0.15	0.883	312	-1.95	0.052	2170
	Component 2	1.28	0.201	357	0.42	0.677	357	0.49	0.628	312	**-2.41**	**0.016**	**2170**
	Component 3	0.04	0.971	357	0.38	0.701	357	-0.18	0.860	312	-1.63	0.104	2170
Thickness	Whole Hipp.	-1.89	0.060	313	1.93	0.055	314	**-3.70**	**0.000**	**258**	**-2.97**	**0.003**	**2350**
	Component 1	-1.42	0.156	747	0.67	0.505	753	**-5.77**	**0.000**	**518**	**-3.36**	**0.001**	**2230**
	Component 2	**-2.01**	**†0.045**	**747**	**2.24**	**†0.025**	**753**	**-2.29**	**0.022**	**518**	-1.33	0.185	2230
	Component 3	-1.26	0.209	747	1.61	0.108	753	-1.73	0.085	518	-0.85	0.398	2230

Table S3 | Statistics for models characterizing cross-sectional and longitudinal associations between hippocampal structure and AD pathology. For each structural measure and each pathological marker (Tau and Amyloid PET), a model was run without exploring component-specific interactions ('Whole Hipp.' Model), for maximum statistical power in detecting non-spatially-specific effects. Then, to characterize such spatial effects, a model was run in which the effects were allowed to vary for each component. The results for each component and for the whole hippocampal model shown in each row. Effects where p<0.05 are shown in bold, with a dagger (†) indicating effects that do not survive correction for multiple comparisons. P-values were calculated from robust model t-values and standard model degrees of freedom (df) (See methods section).

		Tau PET						Amyloid PET					
		Tau (main effect)			Tau * Time			Amyloid (main effect)			Amyloid * Time		
		t-value	p-value	df	t-value	p-value	df	t-value	p-value	df	t-value	p-value	df
R1	Whole Hipp.	**-3.23**	**0.001**	**210**	1.15	0.252	1870	-1.32	0.189	208	0.79	0.429	1850
	Component 1	**-2.77**	**0.006**	**381**	1.81	0.071	1800	-0.86	0.388	373	1.40	0.163	1780
	Component 2	**-2.89**	**0.004**	**381**	0.24	0.814	1800	-1.48	0.139	373	0.62	0.538	1780
	Component 3	**-2.66**	**0.008**	**381**	0.21	0.834	1800	-1.04	0.298	373	-0.54	0.591	1780
MTsat	Whole Hipp.	-0.76	0.446	218	**-3.87**	**0.000**	**1890**	-0.51	0.608	218	-1.21	0.228	1870
	Component 1	-0.10	0.917	432	**-2.32**	**0.021**	**1810**	0.33	0.742	433	-0.68	0.495	1790
	Component 2	-0.79	0.433	432	**-2.18**	**0.029**	**1810**	-0.58	0.560	433	-1.01	0.314	1790
	Component 3	-1.01	0.312	432	**-2.57**	**0.010**	**1810**	-1.05	0.294	433	-0.42	0.673	1790
R2*	Whole Hipp.	-0.15	0.882	214	**-3.10**	**0.002**	**1880**	0.74	0.459	214	**-2.97**	**0.003**	**1860**
	Component 1	1.07	0.286	405	**-2.40**	**0.016**	**1800**	1.59	0.112	411	**-2.38**	**0.018**	**1790**
	Component 2	-0.17	0.863	405	**-2.16**	**0.031**	**1800**	0.62	0.535	411	-1.55	0.121	1790
	Component 3	-1.24	0.216	405	-1.19	0.235	1800	-0.20	0.842	411	-1.62	0.106	1790
PD	Whole Hipp.	0.93	0.352	192	**-4.24**	**0.000**	**1810**	0.84	0.401	193	**-3.03**	**0.003**	**1790**
	Component 1	1.19	0.235	253	**-3.09**	**0.002**	**1770**	0.81	0.420	255	-1.96	0.050	1760

	Component 2	0.99	0.325	253	**-2.66**	**0.008**	**1770**	1.07	0.285	255	**-2.46**	**0.014**	**1760**
	Component 3	0.44	0.661	253	**-2.19**	**0.029**	**1770**	0.42	0.676	255	-1.12	0.265	1760
Thickness	Whole Hipp.	0.15	0.878	237	0.53	0.598	1920	-0.48	0.631	238	-0.52	0.605	1890
	Component 1	-0.17	0.863	552	-1.34	0.180	1830	-0.51	0.614	563	-0.83	0.408	1810
	Component 2	-0.41	0.683	552	1.55	0.122	1830	**-2.02**	**†0.044**	**563**	-0.22	0.827	1810
	Component 3	0.70	0.487	552	0.80	0.425	1830	0.70	0.482	563	-0.01	0.991	1810

Table S4 | Statistics for models characterizing cross-sectional and longitudinal associations between hippocampal structure and APOE4 carrier status. For each structural measure, a model was run without exploring component-specific interactions ('Whole Hipp.' Model), for maximum statistical power in detecting non-spatially-specific effects. Then, to characterize such spatial effects, a model was run in which the effects were allowed to vary for each component. The results for each component and for the whole hippocampal model shown in each row. Effects where p<0.05 are shown in bold, with a dagger (†) indicating effects that do not survive correction for multiple comparisons. P-values were calculated from robust model t-values and standard model degrees of freedom (df).

		APOE4					
		APOE4 (main effect)			APOE4*Time		
		t-value	p-value	df	t-value	p-value	df
R1	Whole Hipp.	**-2.85**	**0.005**	**241**	**3.27**	**0.001**	**2070**
	Component 1	**-2.37**	**0.018**	**420**	**2.15**	**†0.032**	**2000**
	Component 2	**-2.72**	**0.007**	**420**	**2.75**	**0.006**	**2000**
	Component 3	**-2.33**	**0.020**	**420**	1.10	0.270	2000
MTsat	Whole Hipp.	-0.49	0.622	249	**-2.69**	**0.007**	**2090**
	Component 1	0.35	0.730	481	**-2.41**	**0.016**	**2010**
	Component 2	-0.54	0.588	481	-1.53	0.125	2010
	Component 3	-1.00	0.317	481	-1.01	0.312	2010
R2*	Whole Hipp.	**-2.67**	**0.008**	**249**	1.16	0.245	2090
	Component 1	**-2.14**	**0.033**	**479**	1.00	0.316	2010
	Component 2	**-2.40**	**0.017**	**479**	1.22	0.223	2010
	Component 3	**-2.35**	**0.019**	**479**	-0.03	0.976	2010
PD	Whole Hipp.	-0.28	0.780	226	-1.07	0.283	2020
	Component 1	-0.17	0.867	300	-0.67	0.505	1980
	Component 2	-0.05	0.963	300	-1.38	0.167	1980
	Component 3	-0.57	0.570	300	0.09	0.930	1980
Thickness	Whole Hipp.	-1.31	0.191	272	-0.34	0.734	2140
	Component 1	-0.34	0.736	658	0.20	0.843	2040
	Component 2	**-2.08**	**0.038**	**658**	-0.44	0.664	2040
	Component 3	-0.95	0.344	658	-0.42	0.674	2040

Table S5 | Statistics for models characterizing cross-sectional and longitudinal associations between hippocampal structure and delayed recall ability. For each structural measure, a model was run without exploring component-specific interactions ('Whole Hipp.' Model), for maximum statistical power in detecting non-spatially-specific effects. Then, to characterize such spatial effects, a model was run in which the effects were allowed to vary for each component. The results for each component and for the whole hippocampal model shown in each row. Effects where p<0.05 are shown in bold, however no effects survived correction for multiple comparisons within each test (column) (marked with a dagger, †). P-values were calculated from robust model t-values and standard model degrees of freedom (df).

		DR model (longitudinal)						DR * pathology models (baseline)					
		DR (main effect)			DR * Time			DR * Tau			DR * Amyloid		
		t	p	df	t	p	df	t	p	df	t	p	df
R1	Whole Hipp.	**-2.12**	**†0.034**	**1670**	-1.24	0.213	2130	**2.23**	**†0.027**	**181**	**2.72**	**†0.007**	**181**
	Component 1	-1.82	0.070	2080	**-2.26**	**†0.024**	**2100**	1.79	0.075	270	**2.60**	**†0.010**	**269**
	Component 2	-1.58	0.114	2080	-0.27	0.785	2100	1.73	0.085	270	**2.49**	**†0.014**	**269**
	Component 3	-1.49	0.137	2080	-0.10	0.919	2100	**2.47**	**†0.014**	**270**	**2.34**	**†0.020**	**269**
MTsat	Whole Hipp.	0.98	0.329	1440	-0.03	0.977	2080	1.28	0.202	181	**2.22**	**†0.028**	**181**
	Component 1	0.38	0.705	2010	-0.34	0.735	2120	1.26	0.208	277	1.77	0.078	280
	Component 2	1.04	0.301	2010	-0.23	0.818	2120	0.79	0.431	277	**2.20**	**†0.029**	**280**
	Component 3	0.73	0.465	2010	0.43	0.667	2120	1.43	0.155	277	1.85	0.066	280
R2*	Whole Hipp.	0.17	0.869	1480	-1.23	0.218	2090	0.92	0.358	181	1.55	0.124	181
	Component 1	1.09	0.276	2020	-1.91	0.057	2120	1.50	0.136	288	1.96	0.051	294

	Component 2	-1.06	0.290	2020	-0.01	0.988	2120	0.64	0.522	288	1.26	0.210	294
	Component 3	0.22	0.823	2020	-0.58	0.563	2120	0.32	0.751	288	0.97	0.334	294
PD	Whole Hipp.	1.51	0.132	2080	0.20	0.844	2130	1.31	0.193	181	0.86	0.392	181
	Component 1	1.67	0.095	2140	0.30	0.765	2040	1.36	0.175	196	0.94	0.346	197
	Component 2	1.00	0.315	2140	0.02	0.982	2040	1.37	0.171	196	0.87	0.386	197
	Component 3	0.88	0.377	2140	0.09	0.927	2040	1.11	0.266	196	0.71	0.482	197
Thickness	Whole Hipp.	-0.18	0.858	1150	0.58	0.559	1950	**2.02**	**†0.045**	**180**	1.32	0.190	180
	Component 1	1.05	0.296	1910	0.97	0.331	2130	0.86	0.390	497	0.33	0.744	490
	Component 2	-0.10	0.918	1910	-1.09	0.277	2130	**2.04**	**†0.042**	**497**	1.61	0.109	490
	Component 3	-1.21	0.226	1910	0.97	0.335	2130	1.63	0.103	497	1.32	0.187	490

Supporting Information: Sex differences between hippocampal qMRI parameters

Within the age models presented in the main paper, we also tested relationships between biological sex and hippocampal structure. Compared to females, males had significantly lower R1 ($t(253) = -2.76$, $p = 0.006$), lower PD ($t(254) = -2.29$, $p = 0.023$) and greater thickness even after correcting for intracranial volume across the whole hippocampus ($t(260) = 2.20$, $p = 0.028$). The sex differences in R1 and PD were significant in all three components. For thickness, the effect was significant for components 2 only. See Table S6 for full statistics.

Table S6. *Statistics for models characterizing cross-sectional associations between hippocampal structure and sex.* Sex was included as a covariate in the models presented in Fig. 2 and Table 1 in the main paper. The main effect was also allowed to vary by component for the component-varying models. The results for each component and for the whole hippocampal model shown in each row. Statistically significant ($p<0.05$) effects are shown in bold for clarity. P-values were calculated from robust model t-values and standard model degrees of freedom (df).

		t-value	p-value	df
R1	Whole Hipp.	**-2.76**	**0.006**	**253**
	Component 1	**-2.48**	**0.014**	**378**
	Component 2	**-2.55**	**0.011**	**378**
	Component 3	**-2.44**	**0.015**	**378**
MTsat	Whole Hipp.	0.36	0.719	255
	Component 1	0.01	0.992	417
	Component 2	0.19	0.851	417
	Component 3	0.72	0.472	417
R2*	Whole Hipp.	0.12	0.904	257
	Component 1	0.30	0.768	427
	Component 2	-0.07	0.945	427
	Component 3	0.09	0.931	427
PD	Whole Hipp.	**-2.29**	**0.023**	**254**
	Component 1	**-2.42**	**0.016**	**312**
	Component 2	**-2.08**	**0.038**	**312**
	Component 3	**-1.99**	**0.048**	**312**
Thickness	Whole Hipp.	**2.20**	**0.028**	**260**
	Component 1	1.54	0.124	437
	Component 2	**5.29**	**0.000**	**437**
	Component 3	0.03	0.977	437

Supporting Information: Region of no interest does not relate to AD pathology

To infer whether hippocampal structure associations with pathology were a common 'whole brain' feature, we also conducted the same tests in a region on no interest: the precentral gyrus. We did not observe any association between precentral gyrus macro- or micro-structure and pathology measured by PET, either cross-sectionally or longitudinally (Table S7).

Table S7. Statistics for models characterizing cross-sectional and longitudinal associations between region of no interest (Precentral Gyrus) structure and AD pathology

	Tau PET						Amyloid PET					
	Tau (main effect)			Tau * Time			Amyloid (main effect)			Amyloid * Time		
	t-value	p-value	df	t-value	p-value	df	t-value	p-value	df	t-value	p-value	df
R1	-0.65	0.516	240	-1.7	0.091	156	0.636	0.526	239	-1.07	0.288	154
MTsat	-0.576	0.565	239	-0.494	0.622	157	0.767	0.444	239	0.162	0.872	156
R2*	0.0168	0.987	224	-1.23	0.220	149	0.582	0.561	224	-0.422	0.673	147
PD	-1.47	0.143	247	0.901	0.369	151	-0.756	0.451	245	0.581	0.562	149
Thickness	0.861	0.390	207	0.396	0.693	144	-0.932	0.353	208	1.41	0.161	141

Supporting Information: Hippocampal volume associations are in line with those of hippocampal thickness

To enable easier comparison to previous literature, we also ran all models with hippocampal volume as the dependent variable (subfields subiculum and CA1-4). Results are highly similar to all findings regarding hippocampal thickness, with the exception of a significant interaction between delayed recall and amyloid pathology for hippocampal volume which was not statistically significant for hippocampal thickness. Statistics for all models are shown in Table S8.

Table S8. ***Model statistics for hippocampal volume.*** *Each section (1-5) corresponds to a different statistical model, with an equivalent analysis in the main manuscript: 1 (Fig 2), 2-4 (Fig 3), 5 (Fig 4).*

		Age Group		
		t-value	p-value	df
1	Age Group	**-1.97**	**0.0495**	**277**
	Age (Young)	-0.0111	0.991	277
	Age (Older)	**-6.4**	**7.43e-10**	**257**
	Longitudinal change	**-4.87**	**1.42e-06**	**573**
2	Tau (main effect)	-1.03	0.306	203
	Tau * Time	0.0271	0.978	484
3	Amyloid (main effect)	0.419	0.676	203
	Amyloid * Time	-1.24	0.217	477
4	APOE4 (main effect)	-0.841	0.401	239
	APOE4*Time	0.151	0.880	532
5	DR (main effect)	-0.864	0.388	688
	DR * Time	1.07	0.283	603
	DR * Tau	**3.56**	**0.000**	**180**
	DR * Amyloid	**3.46**	**0.001**	**180**

Supporting Information: Hippocampal qMRI does not explain more variance in delayed recall than global PET measures

In order to further explore the relationship between PET, MRI and cognition, we asked whether PET and MRI measures (Averaged across spatial components and hemispheres) jointly explained more variance in delayed recall than either measure alone. To test this, we directly compared the Akaike Information Criteria (AIC) of three models using ANOVA:

PET model: Delayed Recall ~ meta-ROI Tau PET + Global Amyloid PET + age + sex + education

MRI model: Delayed Recall ~ hippocampal structure measure + age + sex + education

Combined model: Delayed Recall ~ hippocampal structure measure + meta-ROI Tau PET + Global Amyloid PET + age + sex + education

The PET model explained 12.7% of the variance of delayed recall, with significant predictors of tau load ($t(295)=-2.07$, $p=0.040$), age ($t(295)=-3.43$, $p<0.001$), sex ($t(295)=-2.16$, $p=0.032$) and education ($t(295)=2.92$, $p=0.004$). MRI models each explained approximately 9% of the delayed recall variance, with no model showing a significant association with delayed recall. The PET model and combined model were both significantly better models (lower AIC) than all MRI models (PET model vs R1 model: $F(296)=11.4$, $p<0.001$; combined model vs R1 model: $F(294)=5.69$, $p=0.004$). The combined model did not offer a significant improvement on the PET model ($F(294)=0.046$, $p=0.831$). These observations are in line with the lack of direct association that we report between delayed recall and MRI measures of the hippocampus (Figure 4).

Supporting Information: Data Distributions

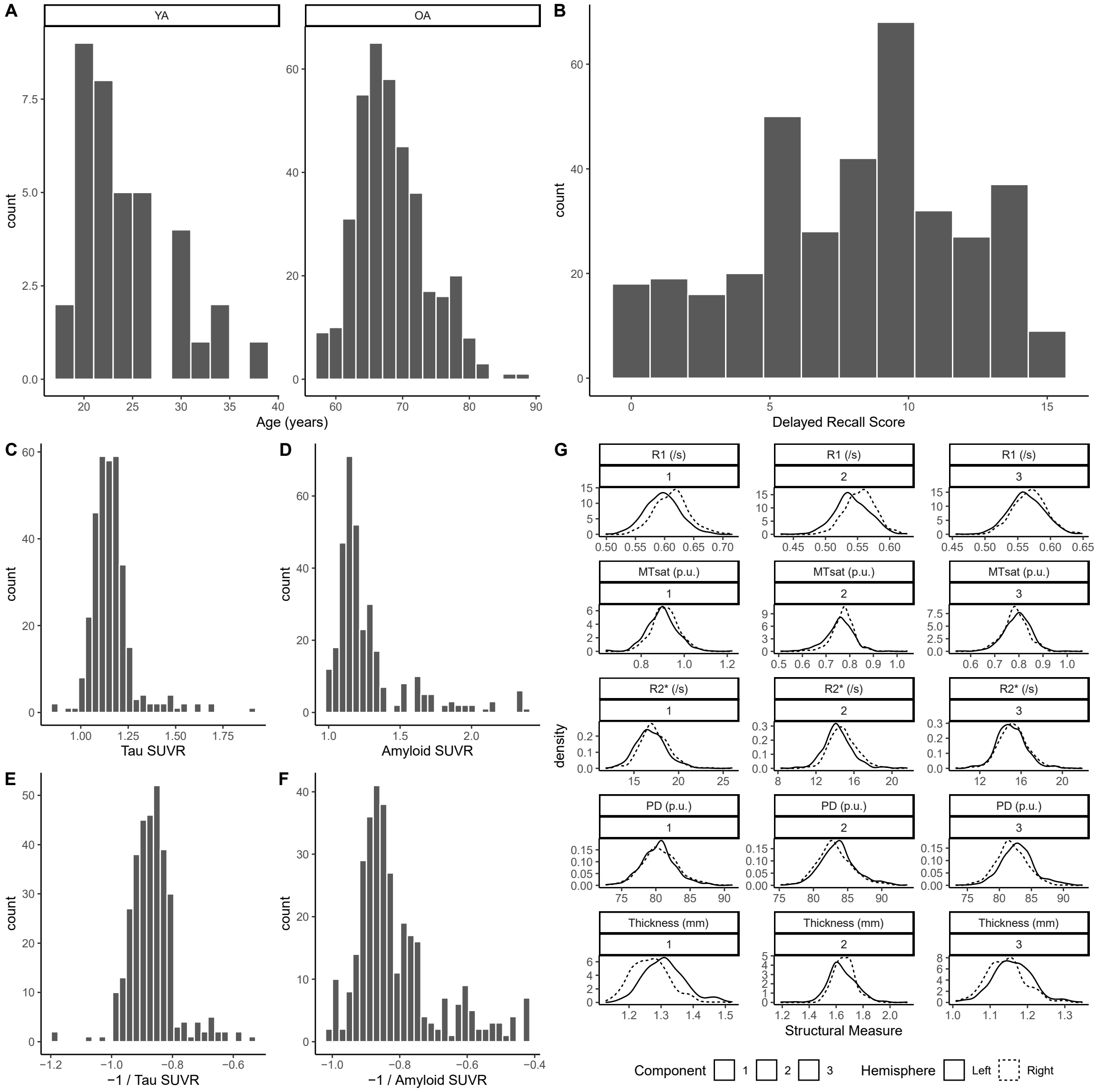

Figure S2. Distributions of variables examined. A) Histograms of baseline age distribution in older (OA) and younger adult (YA) groups. B) Distribution of RAVLT List A Delayed Recall scores, C-F) Distribution of PET-derived measures of pathology expressed as 'raw' SUVR values (C-D) and as transformed values, as used in analyses in the study (E-F). G) Probability Density plots of each of the five structural measures within each component (mapped to colour, as in main manuscript) and also showing separate hemispheres (solid: left, dotted: right).

Supporting Information: Quality of hippocampal segmentation – example subjects

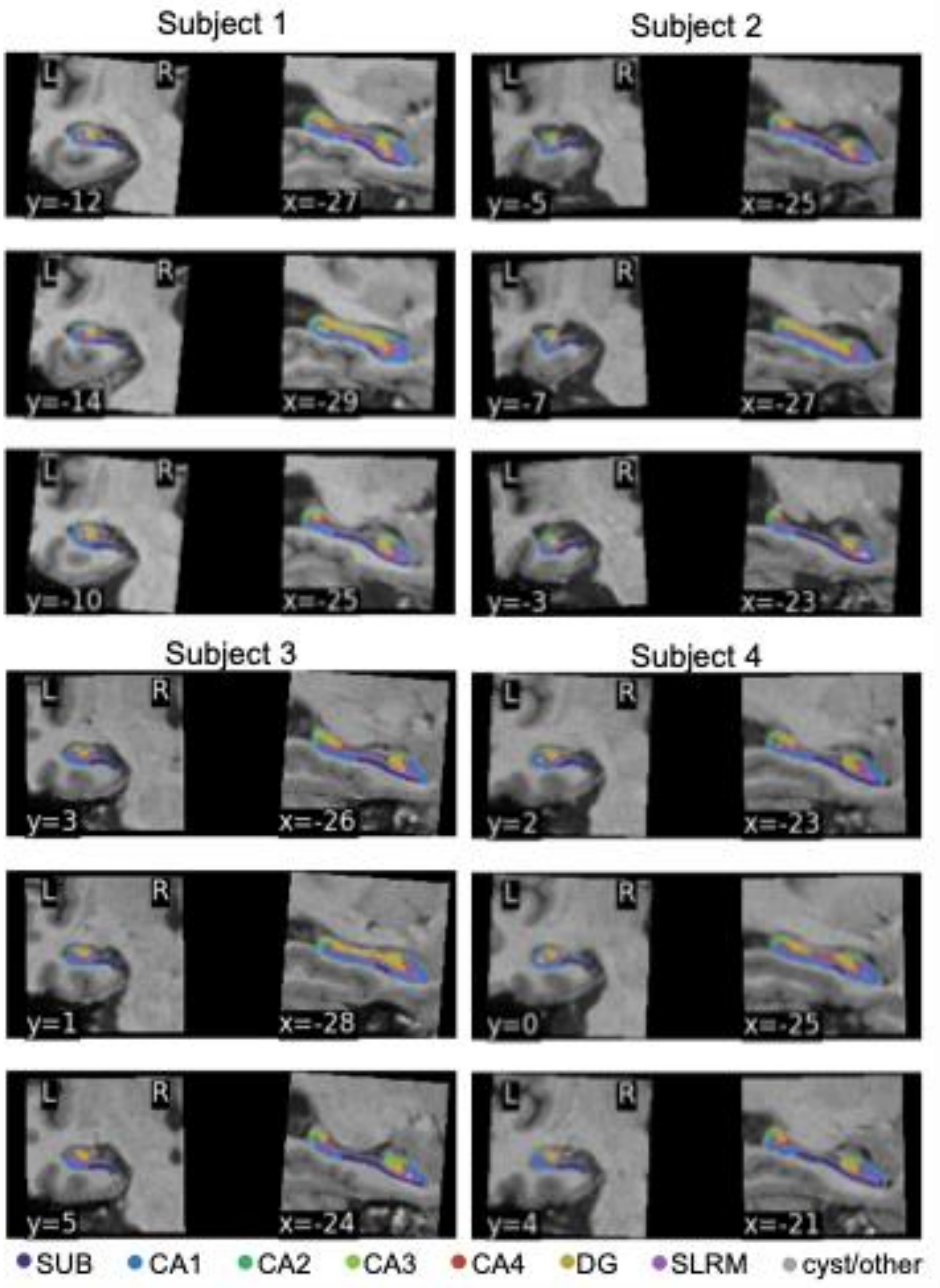

Figure S3. Example hippocampal segmentations created using HippUnfold pipeline. Images show high quality hippocampal segmentation in all cases with respect to variation in individual morphology, including masking of cysts (e.g. Subject 4 y=0 panel). SUB=Subiculum, DG = Dentate Gyrus, SLRM = Stratum lacunosum/radiatum/moleculare (white matter band).

Supporting Information: Reliability analyses

We would ideally be able to calculate within-cohort test-retest reliability measures on all structural measures examined within this study. However, in lieu of multiple datapoints collected within a very short timeframe for our subjects, we have estimated test-retest reliability by comparing left and right hippocampi. Specifically, we have calculated within-subject spatial correlations of left vs right hippocampus, and the vertex-wise consistency (ICC(3,1)) (Figure S4).

We note lower but still reasonable within-subject correlations for microstructural measures as compared to thickness. We also note reasonable and comparable vertex-wise consistency across R1, MTsat and Thickness, with particularly high vertex-wise consistency for PD. R2* has somewhat lower consistency in anterior regions, and may be therefore more sensitive to individual variance in individual folding patterns which are more prominent in the hippocampal head. Asymmetric iron deposits may also drive different variation in R2* signal between hemispheres.

We acknowledge that left vs right comparison is not a perfect test of reliability, given the bias by biological differences between hemispheres – though we have chosen comparison metrics that are agnostic to absolute differences in scale. The largest driver of left-right differences aside from measurement noise is likely to be individual nuance in hippocampal folding patterns on a scale too coarse to reliably delineate at our scan resolution.

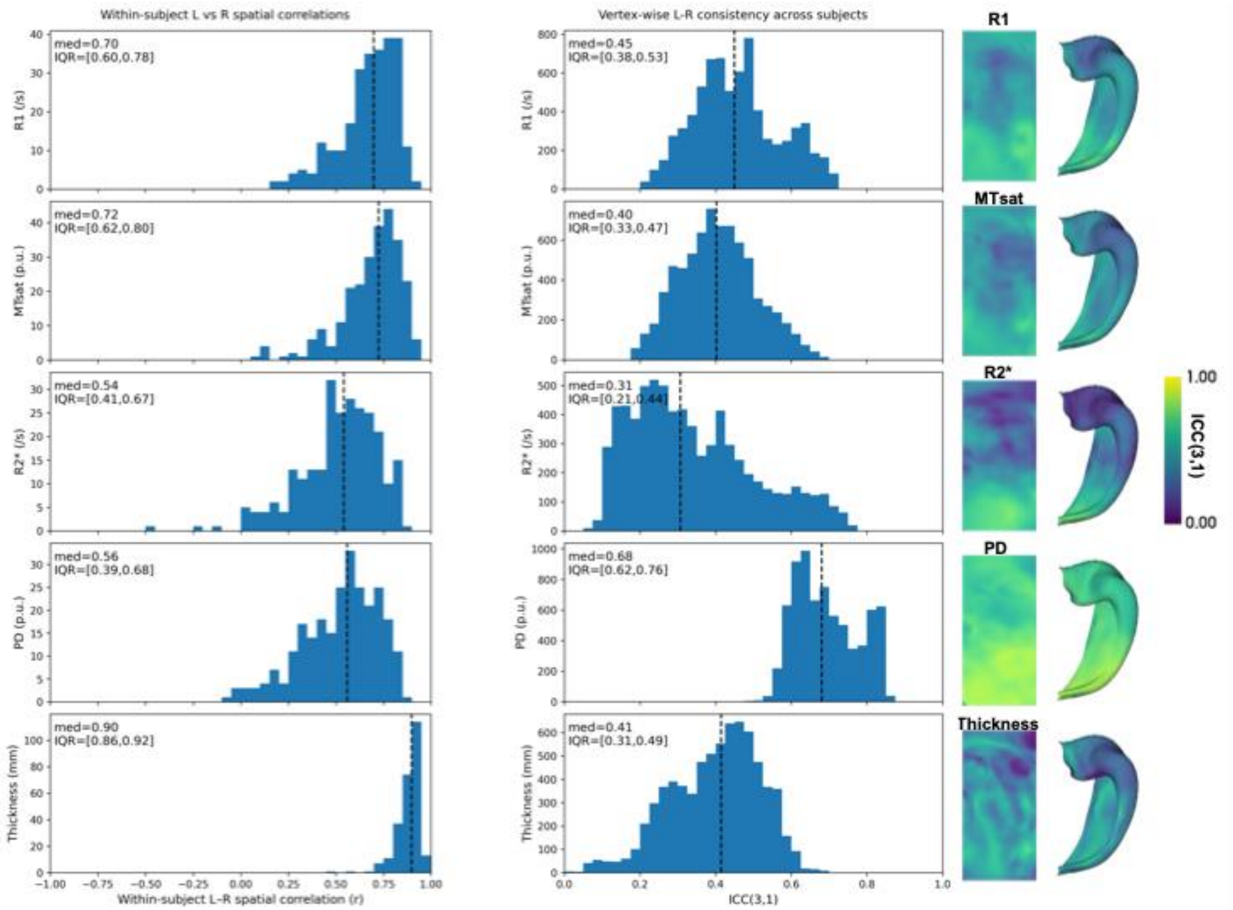

Figure S4 | Reliability metrics for hippocampal structural mapping (Left-Right hemisphere comparison). Left column: Within-subject spatial correlations. Middle/Right columns: ICC(3,1) vertex-wise consistency shown as histogram across vertices (middle) and spatially across the hippocampal surface (right).

Supporting Information: About PREVENT-AD

Full Acknowledgments

PREVENT-AD was launched in 2011 as a $13.5 million 7-year public-private partnership using funds provided by McGill University, the Fonds de Recherche du Québec-Santé (FRQS), an unrestricted research grant from Pfizer Canada, the Levesque Foundation, the Douglas Hospital Research Centre and Foundation, he Government of Canada, the Canada Fund for Innovation, the Canadian Institutes for Health Research, the Alzheimer Society of Canada and the Alzheimer Association. Private sector contributions are facilitated by the Development Office of the McGill University Faculty of Medicine and by the Douglas Hospital Research Centre Foundation (www.douglas.qc.ca).

The primary goal of PREVENT-AD is to test whether serial determination of multi-modal biomarkers of Alzheimer's disease may be measured and used in pre-symptomatic persons at high risk of subsequent AD dementia to trace the progression of the disease process and to measure effects of any potentially preventive treatment interventions. This work is intended to provide preliminary data regarding the probably efficacy and safety of potential new treatments for prevent of AD dementia.

The Founders of the program were John C. S. Breitner, MD, MPH, Judes Poirier, PhD, and Pierre Etienne, MD, Douglas Hospital Research Centre and Faculty of Medicine, McGill University, Montreal, QC, Canada. Program Current Director is Sylvia Villeneuve, PhD, the Co-Director is Judes Poirier, PhD, and the Study Coordinator is Jennifer Tremblay-Mercier, MSc. PREVENT-AD is the result of efforts of many other co-investigators from a range of academic institutions and private corporations, as well as an extraordinarily dedicated and talented clinical and technical assistant staff, students, and postdoctoral fellows. Subjects are recruited from the greater Montreal area and more distant locations in Quebec. For up-to-date information see https://prevent-alzheimer.net/?page_id=42&lang=en

Recruitment Strategy

To bring this population into our program, the main strategy was to send informative flyers about the PREVENT-AD program, in a bag called "publisac" containing different kind of publicity, weekly delivered to the Quebec population. People were invited to contact us by telephone or visit our web site (www.prevent-alzheimer.ca) if they were 55 or older and if they had a parent, brother or sister who has/had Alzheimer's disease. To date, 250,000 flyers were sent in 65 different areas Montreal, QC, CAN. The areas were chosen based on demographic statistics i.e. in areas with higher proportion of 55 years old or older, compared to the provincial average. People were also reached by various media coverage done on the Stop-AD Center and on principal investigators of the Center (television, radio, newspapers). At a smaller level, the program was presented in different academic institutions and nursing homes and flyers were distributed to medical professionals and general population in various occasions.

PREVENT-AD Research Group Authors

The following authors were part of the PREVENT-AD Research Group: Sylvia Villeneuve[1-3], Judes Poirier[1-3], John C.S. Breitner[1-3], Sylvain Baillet[1,4], Andrée-Ann Baril[1-3], Pierre Bellec[1-3,5,6], Véronique Bohbot[1-3], Danilo Bzdok[1], Mallar Chakravarty[1-3], D. Louis Collins[1-4], Mahsa Dadar[1,4], Simon Ducharme[1-4], Alan Evans[1-4], Claudine Gauthier[7], Maiya R. Geddes[1-4,8], Rick Hoge[1-4], Yasser Ituria-Medina[1-4], Gerhard Multhaup[1], Lisa-Marie Münter[1], Alexa Pichet Binette[1,5,6], Natasha Rajah[1-3], Pedro Rosa-Neto[1-3,8], Taylor Schmitz[9], Jean-Paul Soucy[1-4], Nathan Spreng[1,4], Christine Tardif[1-3], Etienne Vachon-Presseau[1,2,10], Christian Bocti[11], Maxime Descoteaux[11], Robert Laforce[12], Pierre Etienne[1-3], Serge Gauthier[1-3,8], Vasavan Nair[1,2,8], Jens Pruessner[1-3], Daniel Auld[1]

1. McGill University, Montreal, QC, CA
2. Douglas Mental Health University Institute Research Centre, Montreal, QC, CA
3. StoP-Alzheimer Centre, Montreal, QC, CA
4. Montreal Neurological Institute and Hospital, Montreal, QC, CA
5. Université de Montréal, Montreal, QC, CA
6. Centre de recherche Institut Universitaire de Gériatrie de Montréal, Montreal, QC, CA
7. Concordia University, Montreal, QC, CA
8. McGill University Research Centre for Studies in Aging, Montreal, QC, CA
9. Western University, London, ON, CA
10. Northwestern University, Chicago, IL, USA
11. Université de Sherbrooke, Sherbrooke, QC, CA
12. Université Laval, Quebec City, QC, CA

SI References

1. Weiskopf N, Suckling J, Williams G, Correia M, Inkster B, Tait R, et al. Quantitative multi-parameter mapping of R1, PD*, MT, and R2* at 3T: a multi-center validation. Frontiers in Neuroscience [Internet]. 2013 [cited 2022 Aug 20];7. Available from: https://www.frontiersin.org/articles/10.3389/fnins.2013.00095

2. Aye N, Lehmann N, Kaufmann J, Heinze HJ, Düzel E, Taubert M, et al. Test-retest reliability of multi-parametric maps (MPM) of brain microstructure. NeuroImage. 2022 Aug 1;256:119249.

3. Tabelow K, Balteau E, Ashburner J, Callaghan MF, Draganski B, Helms G, et al. hMRI - A toolbox for quantitative MRI in neuroscience and clinical research. Neuroimage. 2019 Jul 1;194:191–210.

4. Edwards LJ, Mohammadi S, Pine KJ, Callaghan MF, Weiskopf N. Robust and efficient R2* estimation in human brain using log-linear weighted least squares. In London, United Kingdom; 2022. Available from: https://hdl.handle.net/21.11116/0000-000A-C569-2

5. Filo S, Mezer AA. PD: Proton Density of Tissue Water 1. In: Quantitative MRI of the Brain. 2nd ed. CRC Press; 2018.

6. Mezer A, Rokem A, Berman S, Hastie T, Wandell BA. Evaluating quantitative proton-density-mapping methods. Hum Brain Mapp. 2016 Jun 6;37(10):3623–35.

7. Abbas Z, Gras V, Möllenhoff K, Keil F, Oros-Peusquens AM, Shah NJ. Analysis of proton-density bias corrections based on T1 measurement for robust quantification of water content in the brain at 3 Tesla. Magnetic Resonance in Medicine. 2014;72(6):1735–45.

8. Tofts PS. PD: Proton Density of Tissue Water. In: Quantitative MRI of the Brain [Internet]. John Wiley & Sons, Ltd; 2003 [cited 2023 Feb 8]. p. 83–109. Available from: https://onlinelibrary.wiley.com/doi/abs/10.1002/0470869526.ch4

9. Yamashiro A, Kobayashi M, Saito T. Cerebrospinal fluid T1 value phantom reproduction at scan room temperature. J Appl Clin Med Phys. 2019 Jun 9;20(7):166–75.

10. Hopkins AL, Yeung HN, Bratton CB. Multiple field strength in vivo T1 and T2 for cerebrospinal fluid protons. Magn Reson Med. 1986 Apr;3(2):303–11.

11. Manjón JV, Coupé P, Martí-Bonmatí L, Collins DL, Robles M. Adaptive non-local means denoising of MR images with spatially varying noise levels. J Magn Reson Imaging. 2010 Jan;31(1):192–203.

12. Fischl B. FreeSurfer. Neuroimage. 2012 Aug 15;62(2):774–81.

13. DeKraker J, Haast RA, Yousif MD, Karat B, Lau JC, Köhler S, et al. Automated hippocampal unfolding for morphometry and subfield segmentation with HippUnfold. Forstmann BU, de Lange FP, Bazin PL, editors. eLife. 2022 Dec 15;11:e77945.

14. Isensee F, Jaeger PF, Kohl SAA, Petersen J, Maier-Hein KH. nnU-Net: a self-configuring method for deep learning-based biomedical image segmentation. Nat Methods. 2021 Feb;18(2):203–11.

15. Van Essen DC, Smith SM, Barch DM, Behrens TEJ, Yacoub E, Ugurbil K. The WU-Minn Human Connectome Project: An Overview. Neuroimage. 2013 Oct 15;80:62–79.

16. Braak H, Braak E. Neuropathological stageing of Alzheimer-related changes. Acta Neuropathol. 1991 Sep 1;82(4):239–59.

17. Hyman BT, Hoesen GWV, Damasio AR, Barnes CL. Alzheimer's disease: cell-specific pathology isolates the hippocampal formation. Science. 1984;225(4667):1168–70.

18. Wisse LEM, Chételat G, Daugherty AM, de Flores R, la Joie R, Mueller SG, et al. Hippocampal subfield volumetry from structural isotropic 1 mm3 MRI scans: A note of caution. Hum Brain Mapp. 2021 Feb 1;42(2):539–50.

19. Karat BG, DeKraker J, Hussain U, Köhler S, Khan AR. Mapping the macrostructure and microstructure of the in vivo human hippocampus using diffusion MRI. Human Brain Mapping. 2023;44(16):5485–503.

20. DeKraker J, Cabalo DG, Royer J, Ngo A, Khan AR, Karat BG, et al. HippoMaps: multiscale cartography of human hippocampal organization [Internet]. bioRxiv; 2024 [cited 2024 Dec 17]. p. 2024.02.23.581734. Available from: https://www.biorxiv.org/content/10.1101/2024.02.23.581734v3

21. Sotiras A, Resnick SM, Davatzikos C. Finding imaging patterns of structural covariance via Non-Negative Matrix Factorization. NeuroImage. 2015 Mar 1;108:1–16.

22. Yang Z, Oja E. Linear and Nonlinear Projective Nonnegative Matrix Factorization. IEEE Transactions on Neural Networks. 2010 May;21(5):734–49.

23. Patel R, Steele CJ, Chen AGX, Patel S, Devenyi GA, Germann J, et al. Investigating microstructural variation in the human hippocampus using non-negative matrix factorization. NeuroImage. 2020 Feb 15;207:116348.

24. Gallego-Rudolf J, Wiesman AI, Pichet Binette A, Villeneuve S, Baillet S. Synergistic association of Aβ and tau pathology with cortical neurophysiology and cognitive decline in asymptomatic older adults. Nat Neurosci. 2024 Nov;27(11):2130–7.

25. Jack CR, Wiste HJ, Weigand SD, Therneau TM, Lowe VJ, Knopman DS, et al. Defining imaging biomarker cut-points for brain aging and Alzheimer's disease. Alzheimers Dement. 2017 Mar;13(3):205–16.

26. Desikan RS, Ségonne F, Fischl B, Quinn BT, Dickerson BC, Blacker D, et al. An automated labeling system for subdividing the human cerebral cortex on MRI scans into gyral based regions of interest. Neuroimage. 2006 Jul 1;31(3):968–80.

27. Koller M. robustlmm: An R Package for Robust Estimation of Linear Mixed-Effects Models. Journal of Statistical Software. 2016 Dec 6;75:1–24.

28. Bates D, Mächler M, Bolker B, Walker S. Fitting Linear Mixed-Effects Models Using lme4. Journal of Statistical Software. 2015 Oct 7;67:1–48.
