# Supplementary material for: Quantitative MRI of the hippocampus reveals microstructural trajectories of aging and Alzheimer’s disease pathology
Source: Proc Natl Acad Sci U S A. 2025 Oct 27;122(44):e2502674122. doi: 10.1073/pnas.2502674122 (PMC12595451; doi:10.1073/pnas.2502674122)
Supplement: Supplementary file 2 — Dataset S01 (DOCX) [file pnas.2502674122.sd01.docx]

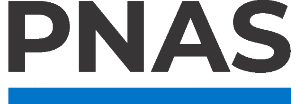


**PREVENT-AD Research Group Authors**

The following authors were part of the PREVENT-AD Research Group: Sylvia Villeneuve^1-3^, Judes Poirier^1-3^, John C.S. Breitner^1-3^, Sylvain Baillet^1,4^, Andrée-Ann Baril^1-3^, Pierre Bellec^1-3,5,6^, Véronique Bohbot^1-3^, Danilo Bzdok^1^, Mallar Chakravarty^1-3^, D. Louis Collins^1-4^, Mahsa Dadar^1,4^, Simon Ducharme^1-4^, Alan Evans^1-4^, Claudine Gauthier^7^, Maiya R. Geddes^1-4,8^, Rick Hoge^1-4^, Yasser Ituria-Medina^1-4^, Gerhard Multhaup^1^, Lisa-Marie Münter^1^, Alexa Pichet Binette^1,5,6^, Natasha Rajah^1-3^, Pedro Rosa-Neto^1-3,8^, Taylor Schmitz^9^, Jean-Paul Soucy^1-4^, Nathan Spreng^1,4^, Christine Tardif^1-3^, Etienne Vachon-Presseau^1,2,10^, Christian Bocti^11^, Maxime Descoteaux^11^, Robert Laforce^12^, Pierre Etienne^1-3^, Serge Gauthier^1-3,8^, Vasavan Nair^1,2,8^, Jens Pruessner^1-3^, Daniel Auld^1^

1. McGill University, Montreal, QC, CA
2. Douglas Mental Health University Institute Research Centre, Montreal, QC, CA
3. StoP-Alzheimer Centre, Montreal, QC, CA
4. Montreal Neurological Institute and Hospital, Montreal, QC, CA
5. Université de Montréal, Montreal, QC, CA
6. Centre de recherche Institut Universitaire de Gériatrie de Montréal, Montreal, QC, CA
7. Concordia University, Montreal, QC, CA
8. McGill University Research Centre for Studies in Aging, Montreal, QC, CA
9. Western University, London, ON, CA
10. Northwestern University, Chicago, IL, USA
11. Université de Sherbrooke, Sherbrooke, QC, CA
12. Université Laval, Quebec City, QC, CA
